# Activation of the cGAS‐STING‐IRF3 Axis by Type I and II Interferons Contributes to Host Defense

**DOI:** 10.1002/advs.202308890

**Published:** 2024-07-14

**Authors:** Zhen Tong, Jia‐Peng Zou, Su‐Yun Wang, Wei‐Wei Luo, Yan‐Yi Wang

**Affiliations:** ^1^ Key Laboratory of Virology and Biosafety Wuhan Institute of Virology Center for Biosafety Mega‐Science Chinese Academy of Sciences Wuhan 430071 China; ^2^ University of Chinese Academy of Sciences Bejing 100049 China; ^3^ Hubei Jiangxia Laboratory Wuhan Hubei 430200 China

**Keywords:** antitumor, antiviral responses, cGAS‐STING‐IRF3, interferon, JAK‐STATs, phosphorylation

## Abstract

Interferons (IFNs) activate JAK‐STAT pathways to induce downstream effector genes for host defense against invaded pathogens and tumors. Here both type I (β) and II (γ) IFNs are shown that can activate the transcription factor IRF3 in parallel with STAT1. IRF3‐deficiency impairs transcription of a subset of downstream effector genes induced by IFN‐β and IFN‐γ. Mechanistically, IFN‐induced activation of IRF3 is dependent on the cGAS‐STING‐TBK1 axis. Both IFN‐β and IFN‐γ cause mitochondrial DNA release into the cytosol. In addition, IFNs induce JAK1‐mediated tyrosine phosphorylation of cGAS at Y214/Y215, which is essential for its DNA binding activity and signaling. Furthermore, deficiency of cGAS, STING, or IRF3 impairs IFN‐β‐ or IFN‐γ‐mediated antiviral and antitumor activities. The findings reveal a novel IRF3 activation pathway parallel with the canonical STAT1/2 activation pathways triggered by IFNs and provide an explanation for the pleiotropic roles of the cGAS‐STING‐IRF3 axis in host defense.

## Graphical abstract

This study shows that both type I and II IFNs can activate the transcription factor IRF3 in parallel with STAT1 through the cGAS‐STING axis, a classic PPR signaling triggered by DNA sensing. Mechanistically, IFN‐caused mtDNA release and JAK1‐mediated phosphorylation of cGAS induce activation of STING‐IRF3 and transcription of effector genes, which contributes to the antiviral and antitumor activities of IFNs.

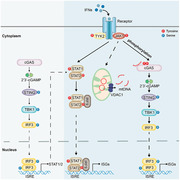

## 1 Introduction

Innate immunity is an intrinsic mechanism of host defense against microbial infections and dysregulated cells.^[^ ^1^ ^]^ The Pattern Recognition Receptors (PRRs) of the innate immune system sense so‐called invaded microbial Pathogen‐Associated Molecular Patterns (PAMPs) or endogenous Danger‐Associated Molecular Patterns (DAMPs), initiating a series of intracellular signaling events that lead to transcriptional induction of type I interferon (IFN) and inflammatory cytokine genes.^[^ ^2^ ^]^ Among the PRRs, the cyclic GMP‐AMP synthase (cGAS) senses both invaded viral DNA and dislocated mitochondrial DNA (mtDNA) or cellular genomic DNA in the cytosol,^[^ ^3^ ^]^ which then uses GTP and ATP as substrates to synthesize cyclic GMP‐AMP (cGAMP).^[^ ^2^, ^4^ ^]^ cGAMP acts as a second messenger molecule to bind to the ER‐associated adaptor protein STING (also called MITA and ERIS),^[^ ^5^ ^]^ which is then translocated from the ER via Golgi apparatus to perinuclear punctate structures.^[^ ^6^ ^]^ During this process, STING recruits the serine/threonine kinase TBK1 and the transcription factor IRF3 to the complex. In the complex, TBK1 first phosphorylates STING, which causes a conformational change of the complex and enables TBK1 to phosphorylate IRF3.^[^ ^7^ ^]^ The phosphorylated IRF3 dimerizes, translocates into the nucleus and binds to the Interferon‐Stimulated Response Element (ISRE) in the promoters of type I IFN and proinflammatory genes, leading to their transcriptional induction.^[^ ^8^ ^]^ Upon RNA virus infection, viral RNA is sensed by the RIG‐I‐like receptors (RLRs) including RIG‐I and MDA5 in the cytosol.^[^ ^9^ ^]^ This initiates a VISA (also called MAVS, IPS, and Cardif)‐TBK1‐dependent signaling cascade that leads to activation of IRF3 and eventual induction of type I IFNs and other antiviral effector genes.^[^ ^10^ ^]^ Infection of certain RNA viruses also causes mitostress to release mtDNA into the cytosol, leading to activation of the cGAS‐STING‐IRF3 axis.^[^ ^11^ ^]^

Type I IFNs, including IFN‐α and IFN‐β, are secreted cytokines that bind to the IFNα/β receptor (IFNAR) consisting of IFNAR1 and IFNAR2 subunits on the cell membrane.^[^ ^12^ ^]^ This binding activates the IFNAR1‐associated tyrosine kinase TYK2 and IFNAR2‐associated tyrosine kinase JAK1, leading to phosphorylation and activation of distinct STAT‐containing transcriptional complexes.^[^ ^13^ ^]^ One classical and important transcriptional complex induced by type I IFNs is the ISG factor 3 (ISGF3) complex, which consists of tyrosine‐phosphorylated STAT1, STAT2, and unphosphorylated IRF9.^[^ ^14^ ^]^ The ISGF3 complex binds to ISRE in the promoters of a set of downstream antiviral genes called Interferon Stimulated Genes (ISGs).^[^ ^13b^ ^]^ Other transcriptional complexes induced by type I IFNs contain homodimers of STATs such as STAT1, 2, and 3, which bind to the IFN‐γ‐Activated Site (GAS) elements in the promoters of certain ISGs.^[^ ^15^ ^]^ Type I IFNs induce the expression of hundreds of ISGs, which mediate various biological responses including antiviral effects.^[^ ^14^, ^16^ ^]^ However, it has been well demonstrated that the canonical JAK‐STAT pathways are not sufficient to account for all the pleiotropic effects of type I IFNs and other signaling cascades are important for complementing their roles. For example, it has been demonstrated that activation of the p38 kinase cascade is important for type I IFN‐induced expression of ISG15 as well as antiviral and anti‐proliferative effects.^[^ ^17^ ^]^

Type II IFN contains the sole IFN‐γ, which is produced mostly by immune cells and also exhibits antiviral and anti‐proliferative effects.^[^ ^13^, ^18^ ^]^ IFN‐γ binds to IFNGR, a receptor that consists of IFNGR1 and IFNGR2 subunits. Binding of IFN‐γ induces phosphorylation of IFNGR1‐associated JAK1 and IFNGR2‐associated JAK2, leading to their phosphorylation and activation of STAT1 in most cells as well as STAT2 and STAT3 in certain cells.^[^ ^13a^ ^]^ The activated STAT1‐STAT1 homodimers bind to the GAS elements in the promoters of downstream effects genes, leading to their transcriptional induction and antiviral and anti‐proliferative effects.^[^ ^13a^ ^]^ Similar to type I IFNs, the canonical JAK‐STAT pathways are also not sufficient to account for all the IFN‐γ‐triggered biological effects.^[^ ^19^ ^]^ Whether other signaling cascades are involved in the shared antiviral and anti‐proliferative effects of type I and II IFNs is unknown.

In this study, we show both type I and II IFNs can activate the transcription factor IRF3 in parallel with STAT1/2. IRF3‐deficiency does not affect JAK‐STAT activation but impairs the transcription of a subset of ISGs induced by both type I and II IFNs. IFN‐β and IFN‐γ cause mtDNA release into the cytosol and JAK1‐mediated phosphorylation of cGAS, leading to activation of the cGAS‐STING‐IRF3 axis to induce transcription of a set of ISRE‐dependent effector genes. We further show that deficiency of cGAS, STING, and IRF3 impairs IFN‐mediated antiviral and antitumor activities. Our findings suggest that the cGAS‐STING‐IRF3 axis is an important arm of IFN‐induced pathways and provides an explanation of the pleiotropic roles of the cGAS‐STING‐IRF3 axis in host defense.

## 2 Results

### 2.1 IFN‐β  and IFN‐γ Induce IRF3‐dependent Transcription of Downstream Effector Genes

**Figure 1:**
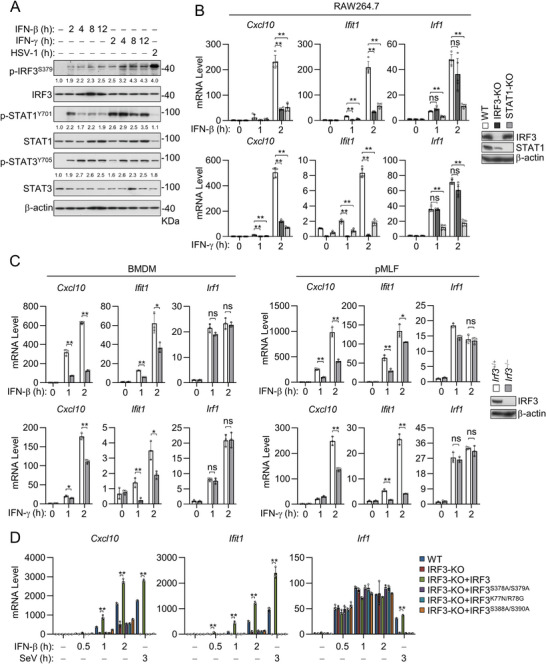
IFN‐β  and IFN‐γ induce IRF3‐dependent transcription of downstream effector genes. A) IFN‐β and IFN‐γ induce activation of IRF3. Immunoblot analysis of the indicated proteins in MLFs stimulated with IFN‐β (20 ng mL^−1^), IFN‐γ (20 ng mL^−1^), or infected with HSV‐1 (MOI = 1) for the indicated times. The relative quantification of the indicated proteins is shown as the mean of 2 independent experiments. B) Effects of knockout of IRF3 and STAT1 on IFN‐β‐ and IFN‐γ‐induced transcription of downstream genes in RAW264.7. qPCR analysis of mRNA abundance of the indicated genes in IRF3‐ or STAT1‐deficient RAW264.7 cells un‐treated or treated with IFN‐β (20 ng mL^−1^) or IFN‐γ (20 ng mL^−1^) for the indicated times. Knockout efficiency of STAT1 and IRF3 in RAW264.7 was analyzed by immunoblotting with the indicated antibodies. Data are shown as mean ± SD (*n* = 5), and statistical significance was determined by one‐way ANOVA; **p < 0.01. ns, not significant. C) Effects of knockout of IRF3 on IFN‐β‐ or IFN‐γ‐induced transcription of downstream genes. qPCR analysis of mRNA abundance in *Irf3* ^+/+^ and *Irf3* ^−/−^ BMDMs and pMLFs unstimulated or stimulated with IFN‐β (20 ng mL^−1^ ) or IFN‐γ (20 ng mL^−1^) for the indicated times. Knockout efficiency of IRF3 pMLFs was analyzed by immunoblotting with the indicated antibodies. Data are shown as mean ± SD (*n* = 3), and statistical significance was determined by unpaired two‐tailed Student‘s *t‐*test. *p < 0.05; **p < 0.01. ns, not significant. D) Effects of IRF3 mutants on IFN‐β‐induced transcription of downstream genes. IRF3‐deficient RAW264.7 cells reconstituted with IRF3 or the indicated IRF3 mutants were treated with IFN‐β (20 ng mL^−1^) for the indicated times before qPCR analyzed the transcription of downstream genes. Data are shown as mean ± SD (*n* = 3), and statistical significance was determined by one‐way ANOVA. **p < 0.01.

Previously, it has been well established that the transcription factor IRF3 is required for PRR‐mediated induction of type I IFNs, which further induce downstream innate immune effector genes.^[^ ^20^ ^]^ However, whether IRF3 is activated by IFNs and required for amplification of the expression of downstream ISGs is unclear. To investigate these questions, we first examined whether stimulation of IFNs induces phosphorylation and activation of IRF3. As expected, IFN‐β and IFN‐γ induced phosphorylation of STAT1^Y701^ and STAT3^Y705^ in mouse lung fibroblasts (MLFs), which is a hallmark of mouse STAT1/STAT3 activation.^[^ ^21^ ^]^ (**Figure** **1**a). In these experiments, IFN‐α/β and IFN‐γ also induced phosphorylation of mouse IRF3^S379^ (equivalent to human IRF3^S386^), which is a hallmark of IRF3 activation^[^ ^8b^ ^]^ (Figure 1A; Figure S1A, Supporting Information). In addition, dimerization and nuclear translocation of IRF3 were induced after IFN‐β treatment (Figure S1B,C, Supporting Information). These results suggest that IRF3 is activated by both type I and II IFNs. Intriguingly, phosphorylated IRF7^S437/S438^ was also increased after IFN‐β but not IFN‐γ treatment, which was largely due to the induced expression of IRF7 (Figure S1D, Supporting Information). IFN‐β or IFN‐γ did not induce transcription of *IFNB1* and *IFNA* genes that require synergistic activation of NF‐κB, IRF3/7, and AP‐1 (Figure S1E, Supporting Information). It has been previously reported that IFN‐γ induces IRF3 phosphorylation and IFN‐β expression,^[^ ^22^ ^]^ prompting us to investigate whether the effects of IFN‐γ on IRF3 activation are mediated by the IFN‐β loop. We found that pre‐treatment with an IFNAR‐blocking antibody showed little effect on IFN‐γ‐triggered IRF3 phosphorylation (Figure S1F, Supporting Information), suggesting the IFN‐β loop barely plays a role in the activation of IRF3 induced by IFN‐γ.

To investigate the roles of endogenous IRF3 in IFN‐induced signaling, we generated IRF3‐deficient cells. qPCR experiments indicated that knockout of IRF3 in murine macrophage RAW264.7 cells by CRISPR‐Cas9 inhibited IFN‐β‐ and IFN‐γ‐induced transcription of downstream *Cxcl10* and *Ifit1* genes (which contain ISRE in their promoters.^[^ ^23^ ^]^) but not the *Irf1* gene (which contains GAS in its promoter.^[^ ^23^ ^]^) (Figure 1B). In these experiments, knockout of STAT1 inhibited transcription of all examined downstream genes including *Cxcl10*, *Ifit1*, and *Irf1* induced by IFN‐β and IFN‐γ (Figure 1B). Similarly, IFN‐β‐ and IFN‐γ‐triggered induction of *Cxcl10* and *Ifit1* but not *Irf1* genes was impaired in *Irf3* ^−/−^ mouse bone marrow‐derived macrophages (BMDMs) and primary MLFs (pMLFs) in comparison with their wild‐type counterparts (Figure 1C), revealing an essential role of IRF3 in both primary and immortalized cells. Additionally, knockdown of IRF3 by siRNA inhibited IFN‐β and IFN‐γ‐induced transcription of *Cxcl10*, *Ifit1* but not *Irf1* in primary human foreskin fibroblasts (pHFFs) (Figure S2A, Supporting Information). qPCR analysis of more ISG genes confirmed that IRF3‐deficiency impeded IFN‐β/γ−induced transcription of multiple ISRE‐containing ISGs (Figure S2B, Supporting Information). These results suggest that IRF3 is required for induction of the ISRE‐containing genes induced by both type I and II IFNs in both murine and human cells.

Previously, it has been demonstrated that IRF3 is activated by TBK1‐mediated phosphorylation, which then dimerizes and translocates from the cytoplasm to the nucleus where it binds to ISRE to induce transcription of type I IFN and other downstream effector genes.^[^ ^8b^ ^]^ We reconstituted IRF3‐deficient RAW264.7 cells with wild‐type IRF3, IRF3^S378A/S379A^ (a mutant that can not be dimerized), IRF3^K77N/R78G^ (a mutant that is unable to translocate into the nucleus), and IRF3^S388A/S390A^ (a mutant lacking transcriptional activity).^[^ ^24^ ^]^ qPCR experiments indicated that reconstitution of wild‐type IRF3 but not the examined mutants rescued IFN‐β‐induced transcription of *Cxcl10* and *Ifit1* genes in IRF3‐deficient cells (Figure 1D). In these experiments, IRF3‐deficiency and reconstitution with wild‐type IRF3 or its mutants had no marked effects on IFN‐β‐induced transcription of *Irf1* gene (Figure 1D). These data suggest that IRF3 phosphorylation and transcriptional activity are required for IFN‐triggered induction of a set of ISRE‐dependent downstream genes.

**Figure 2:**
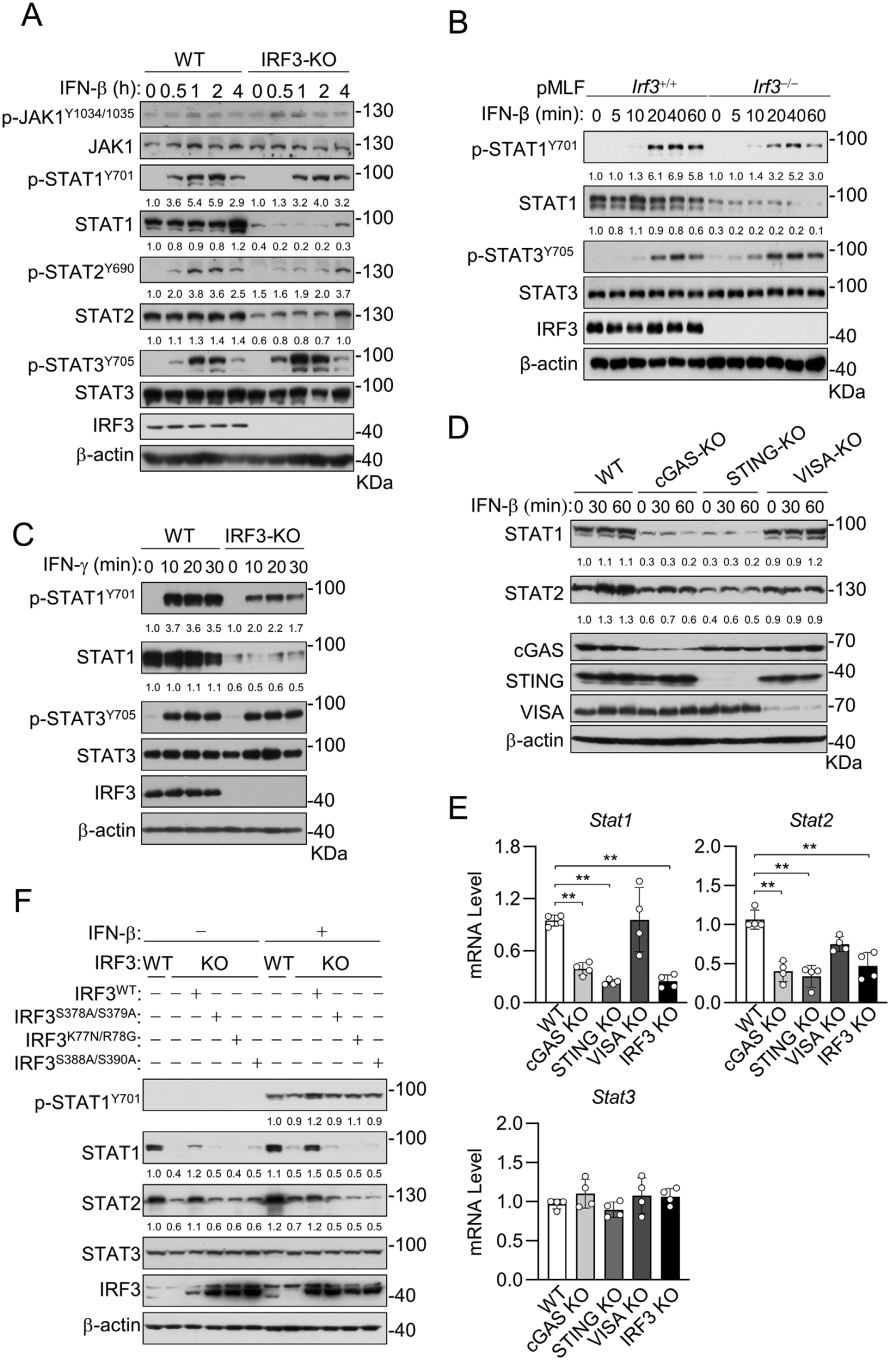
The cGAS‐STING‐TBK1‐IRF3 axis is important for maintaining basal STAT1/2 expression. A) Effects of knockout of IRF3 on IFN‐β‐induced signaling in RAW264.7. Immunoblot analysis of the indicated proteins in WT and IRF3‐deficient RAW264.7 cells was untreated or treated with IFN‐β (20 ng mL^−1^) for the indicated times. B) Effects of knockout of IRF3 on IFN‐β‐induced signaling in pMLFs. Immunoblot analysis of the indicated proteins in *Irf3* ^+/+^ and *Irf3* ^−/−^ pMLFs was untreated or treated with IFN‐β (20 ng mL^−1^) for the indicated times. The relative amount of the indicated blot bands was quantified by densitometry with Image J software and normalized to the control. C) Effects of knockout of IRF3 on IFN‐γ‐induced signaling in RAW264.7. Immunoblot analysis of the indicated proteins in WT and IRF3‐deficient RAW264.7 cells untreated or treated with IFN‐γ (20 ng mL^−1^) for the indicated times. D) Effects of knockout of cGAS, STING, or VISA on IFN‐β‐induced signaling in RAW264.7 cells. Immunoblot analysis of the indicated proteins in WT, cGAS‐, STING‐ or VISA‐deficient RAW264.7 cells untreated or treated with IFN‐β (20 ng mL^−1^) for the indicated times. E) Knockout of cGAS, STING, and IRF3 but not VISA reduces basal mRNA levels of *Stat1* and *Stat2*. qPCR analysis of mRNA abundance of the indicated genes in WT, cGAS‐, STING‐, VISA‐, or IRF3‐deficient RAW264.7 cells. Data are shown as mean ± SD (*n* = 4), and statistical significance was determined by unpaired two‐tailed Student‘s *t‐*test. **p < 0.01. F) Reconstitution of IRF3 but not its inactive mutants rescues the expression of STAT1 and STAT2 in IRF3‐deficient MLF cells. WT or IRF3‐KO cells reconstituted with the indicated plasmids were untreated or treated with IFN‐β (20 ng mL^−1^) for 2 h before being analyzed by immunoblotting with the indicated antibodies. The relative quantification of the indicated proteins is shown in (A‐D&F) expressed as the mean of 2 independent experiments.

Biochemically, IRF3‐deficiency had no marked effects on IFN‐β‐induced phosphorylation of JAK1^Y1034/1035^ (p‐JAK1^Y1034/1035^) or STAT3^Y705^ (p‐STAT3^Y705^) (which are hallmarks of their activation) as well as total levels of JAK1 and STAT3 in RAW264.7 cells (**Figure** **2**a). Interestingly, IRF3‐deficiency caused dramatic down‐regulation of total STAT1 and STAT2 in un‐stimulated and IFN‐β‐stimulated RAW264.7 cells, but only caused minimal down‐regulation of phosphorylated STAT1^Y701^ (p‐STAT1^Y701^) and STAT2^Y690^ (p‐STAT2^Y690^) following IFN‐β stimulation (Figure 2A; Figure S3, Supporting Information). Similar results were also observed with primary MLFs (Figure 2B). Consistently, type II interferon also caused dramatic down‐regulation of STAT1 and a relatively minor decrease of p‐STAT1^Y701^ in RAW264.7 cells (Figure 2C). These results suggest that IRF3 is important for maintaining the basal levels of STAT1 and STAT2 but not required for their phosphorylation and activation per se.

We further investigated how IRF3 deficiency causes down‐regulation of STAT1/2 but not STAT3. We first investigated whether the known upstream components, including cGAS and STING in the DNA‐triggered pathway and VISA in the viral RNA sensor RIG‐I/MDA5‐triggered pathway, are also required for maintaining STAT1/2 stability. We found that knockout of cGAS and STING but not VISA caused down‐regulation of STAT1 and STAT2 in un‐stimulated as well as IFN‐β‐stimulated cells (Figure 2D). These results suggest that basal activation of the cGAS‐STING‐IRF3 axis is important for maintaining STAT1/2 at proper levels. Further experiments indicated that knockout of cGAS, STING or IRF3 markedly down‐regulated mRNA of *Stat1* and *Stat2* but not *Stat3*, whereas knockout of VISA had minimal effects on the mRNA levels of *Stat1*, *Stat2*, and *Stat3* genes (Figure 2E). Consistently, reconstitution with wild‐type IRF3 but not its transcriptionally inactive mutants rescued the protein level of STAT1/2 in IRF3‐deficient MLF cells (Figure 2F). In these experiments, the reconstitution of wild‐type IRF3 did not further increase the level of p‐STAT1^Y701^ (Figure 2F). Taken together, these results suggest that cGAS‐STING‐mediated basal activation of IRF3 is important for transcriptional induction of STAT1/2 under physiological conditions. This is consistent with previous studies which demonstrate STAT1/2 are induced by IFNs.^[^ ^21^ ^]^

### 2.2 IFNs Induce IRF3 Activation via the cGAS‐STING Axis

**Figure 3:**
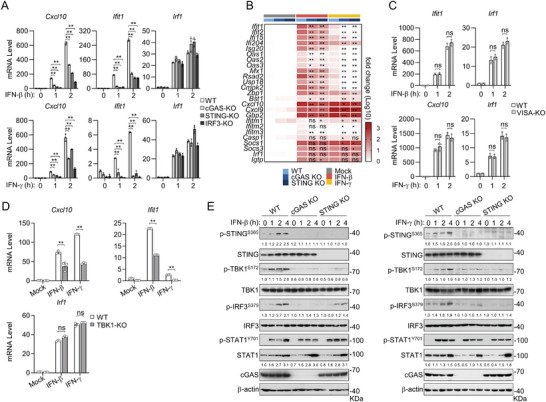
IFNs induce IRF3 activation via the cGAS‐STING axis. A) Effects of knockout of cGAS, STING or IRF3 on IFN‐β‐ and IFN‐γ‐induced transcription of downstream genes in RAW264.7. qPCR analysis of mRNA abundance of the indicated genes in WT, cGAS‐, STING‐, and IRF3‐deficient RAW264.7 stimulated with IFN‐β (20 ng mL^−1^) or IFN‐γ (20 ng mL^−1^) for the indicated times. B) A heat map of qPCR of the indicated genes from WT, cGAS‐, and STING‐deficient RAW264.7 stimulated with IFN‐β (20 ng mL^−1^) or IFN‐γ (20 ng mL^−1^) for 2 h. The scale bar represents the fold change of mRNA abundance of each gene (log10 value). C) Effects of VISA knockout on IFN‐β‐ and IFN‐γ‐induced transcription of downstream genes in RAW264.7 cells. qPCR analysis of mRNA abundance of the indicated genes in WT or VISA‐deficient RAW264.7 cells un‐treated or treated with IFN‐β (20 ng mL^−1^) or IFN‐γ (20 ng mL^−1^) for the indicated times. D) Effects of TBK1 knockout on IFN‐β‐and IFN‐γ‐induced transcription of downstream genes in RAW264.7 cells. qPCR analysis of mRNA abundance of the indicated genes in WT or TBK1‐deficient RAW264.7 cells un‐treated or treated with IFN‐β (20 ng mL^−1^) or IFN‐γ (20 ng mL^−1^) for 1 h. E) Effects of knockout of cGAS and STING on IFN‐β‐ or IFN‐γ‐induced JAK‐STAT and IRF3 activation. Immunoblot analysis of the indicated proteins in WT, cGAS‐ and STING‐deficient RAW264.7 cells un‐treated or treated with IFN‐β (40 ng mL^−1^) or IFN‐γ (40 ng mL^−1^) for the indicated times. The relative quantification of the indicated proteins is shown as the mean of two independent experiments. Data are shown as mean ± SD (n = 3), and statistical significance was determined by one‐way ANOVA (A, B) or unpaired two‐tailed Student‘s *t‐*test (C, D). *p < 0.05; **p < 0.01; ns, not significant.

Since IRF3 activity is required for IFN‐triggered induction of a subset of downstream effector genes such as *Cxcl10*, *Ifit1*, *Stat1*, and *Stat2*, we investigated how IFNs activate IRF3. Previously, it has been reported that both the DNA‐sensing cGAS‐STING and the viral RNA‐sensing RLR‐VISA axes activate the kinase TBK1 to phosphorylate and activate the transcription factor IRF3.^[^ ^2a^ ^]^ Therefore, we examined whether these signaling axes are involved in IFN‐triggered IRF3 activation. We found that knockout of either cGAS or STING markedly inhibited IFN‐β‐ and IFN‐γ‐triggered induction of *Cxcl10* and *Ifit1* but not *Irf1* genes in RAW264.7 cells (**Figure** **3**a). We also examined the effects of knockout of cGAS or STING on transcription of more ISG genes induced by IFN‐β and IFN‐γ. The results indicated that transcription of multiple ISRE‐containing ISGs was inhibited in cGAS‐ or STING‐deficient cells (Figure 3B). In similar experiments, knockout of VISA had no marked effects on IFN‐β‐triggered induction of *Ifit1* and *Irf1* genes or IFN‐γ‐triggered induction of *Cxcl10* and *Irf1* genes in RAW264.7 cells (Figure 3C), whereas knockout of TBK1 (which is a kinase for IRF3) inhibited IFN‐β or IFN‐γ‐triggered induction of *Cxcl10* and *Ifit1* but not *Irf1* gene (Figure 3D). These results suggest that the cGAS‐STING‐TBK1‐IRF3 axis plays a role in the IFN‐induced transcription of ISRE‐containing ISGs.

Biochemically, knockout of cGAS inhibited phosphorylation of its downstream components STING^S365^, TBK1^S172^ and IRF3^S379^ (which are hallmarks for their respective activation,^[^ ^1a^ ^]^ whereas knockout of STING inhibited phosphorylation of its downstream components TBK1^S172^ and IRF3^S379^ induced by stimulation with either IFN‐β or IFN‐γ in RAW264.7 (Figure 3E). In these experiments, knockout of cGAS and STING down‐regulated basal STAT1 levels but had no marked effects on the phosphorylation of STAT1^Y701^ following IFN‐β or IFN‐γ stimulation (Figure 3E). These results further support the conclusion that IFN‐β and IFN‐γ activate the cGAS‐STING‐TBK1‐IRF3 axis to induce transcription of a set of downstream genes.

### 2.3 IFNs Trigger mtDNA Release into the Cytosol

**Figure 4:**
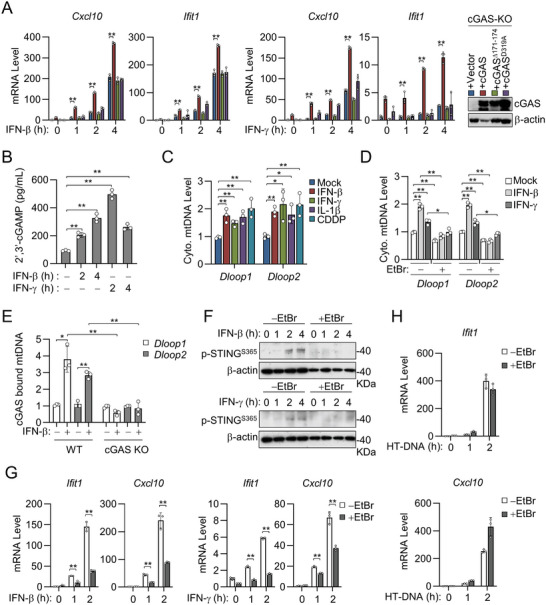
IFNs induce mtDNA release into the cytosol to activate cGAS. A) Effects of reconstitution of cGAS or its mutants on IFN‐β‐ or IFN‐γ‐induced transcription of downstream genes in cGAS‐deficient cells. qPCR analysis of the indicated mRNA abundance in cGAS‐knockout MLFs reconstituted with cGAS or its mutants and stimulated with IFN‐β (20 ng mL^−1^) or IFN‐γ (20 ng mL^−1^) for the indicated times. Expression of cGAS and the indicated mutants in the reconstituted cells was confirmed by immunoblotting analysis. B) Measurement of cGAMP production in RAW264.7 after IFN‐β and IFN‐γ stimulation. RAW264.7 were treated with IFN‐β (40 ng mL^−1^) or IFN‐γ (40 ng mL^−1^) for the indicated times. Cell lysates were collected and analyzed by cGAMP ELISA. C) Measurement of cytosolic mtDNA genes in MLFs after IFN‐β and IFN‐γ stimulation. DNA was harvested from cytosolic and whole cell lysate of MLFs treated with IFN‐β (40 ng mL^−1^, 3 h), IFN‐γ (40 ng mL^−1^, 3 h), IL‐1β (10 ng mL^−1^, 9 h), or CDDP (25 µM, 9 h). Quantification of mtDNA in the cytosolic fraction was performed by qPCR using *Dloop1* and *Dloop2* genes and normalized to the nuclear gene *Tert* in the total fraction. Data are presented as fold enrichment over media‐treated controls. D) EtBr treatment decreases levels of cytosolic mtDNA in MLFs. MLFs were left untreated or treated for 14 days in a medium containing EtBr (100 ng mL^−1^) to deplete mtDNA. EtBr‐treated and untreated MLFs were stimulated with IFN‐β (20 ng mL^−1^) or IFN‐γ (20 ng mL^−1^) for 4 h. Quantification of mtDNA was performed by qPCR using *Dloop1* and *Dloop2* genes present in the cytosolic fraction and normalized to the nuclear gene *Tert* in the total fraction. Data are presented as fold enrichment over media‐treated controls. E) IFN‐β stimulation increases the abundance of mtDNA bound to cGAS. WT and cGAS‐knockout MLFs were unstimulated or stimulated with IFN‐β (20 ng mL^−1^). Cell lysates were collected and immunoprecipitated with cGAS antibody, followed by qPCR analysis of extracted DNA to detect cGAS‐bound mtDNA abundance. The fold enrichment of mtDNA in cGAS immunoprecipitates in cells treated with IFN‐β was calculated as compared with that in un‐stimulated cells. F)EtBr treatment inhibits IFN‐β and IFN‐γ‐induced phosphorylation of STING. Untreated or EtBr‐treated cells were stimulated with IFN‐β (40 ng mL^−1^) or IFN‐γ (40 ng mL^−1^) for the indicated times before being analyzed by immunoblotting with the indicated antibodies. G) EtBr treatment inhibits IFN‐β and IFN‐γ‐induced transcription of downstream *Ifit1* and *Cxcl10* genes. qPCR analysis of the indicated genes from EtBr‐treated and untreated MLFs stimulated with IFN‐β (20 ng mL^−1^) or IFN‐γ (20 ng mL^−1^) for the indicated times. H) EtBr has no effects on HT‐DNA‐induced transcription of downstream *Ifit1* and *Cxcl10* genes. qPCR analysis of the indicated genes from EtBr‐treated and untreated MLFs transfected with HT‐DNA (1 µg mL^−1^) for the indicated times. Data are shown as mean ± SD (*n* = 3), and statistical significance was determined by one‐way ANOVA (A, C, D) or unpaired two‐tailed Student‘s *t‐*test (B, E, G). *p < 0.05; **p < 0.01.

We next investigated how the cGAS‐STING axis is activated following IFN stimulation. Since cGAS is activated by sensing cytosolic DNA, we hypothesized that IFN stimulation causes the release of mitochondrial DNA (mtDNA) into the cytosol, which is sensed by cGAS. To test this, we reconstituted cGAS‐deficient MLFs with wild‐type cGAS, the DNA‐binding deficient mutant cGAS^Δ171−174^, or the enzymatic inactive mutant cGAS^D319A [^ ^25^ ^]^ and measured their effects on IFN‐triggered induction of downstream effector genes. qPCR experiments indicated that reconstitution with cGAS but not the examined mutants increased IFN‐β‐ and IFN‐γ‐induced transcription of *Cxcl10* and *Ifit1* genes (**Figure** **4**a). Consistently, IFN‐β and IFN‐γ treatment led to the production of cGAMP (Figure 4B). Taken together, these findings suggest that the function of cGAS in IFN‐triggered signaling is dependent on its DNA‐binding and enzymatic activities.

We next investigated whether IFN stimulation causes the release of mtDNA. Using qPCR to quantitate cytosolic mtDNA *D‐loop* gene in MLFs, we found that IFN‐β and IFN‐γ caused the release of mtDNA into the cytosol (Figure 4C), which inhibited the following mtDNA elimination by ethidium bromide (EtBr) (Figure 4D). In addition, IFN‐β treatment induced a significant increase of mtDNA bound to cGAS, which was eliminated in cGAS‐deficient cells (Figure 4E). Depletion of mtDNA by EtBr inhibited IFN‐β‐ and IFN‐γ‐induced phosphorylation of STING (Figure 4F) as well as induction of downstream *Ifit1* and *Cxcl10* genes (Figure 4G). In contrast, EtBr treatment showed no effects on transcription of downstream genes induced by transfected HT‐DNA (Figure 4H). These results suggest that IFN‐β‐ and IFN‐γ trigger mtDNA release into the cytosol, which is required for activation of cGAS‐STING signaling.

We next investigated the mechanisms of mtDNA release induced by IFNs. Previously, it has been demonstrated that mitochondrial outer membrane permeabilization (MOMP) is required for mtDNA release. Both BAX/BAK oligomers and VDAC oligomers can form large macropores in the mitochondrial outer membrane (MOM) and mediate mtDNA release.^[^ ^26^ ^]^ While mtDNA release via BAX/BAK has been shown to be related to cell death, VDAC‐mediated mtDNA release is milder and has been shown to induce the production of IFNs via cGAS.^[^ ^27^ ^]^

We then investigated the involvement of cell death and mitochondrial dysfunction in the release of mtDNA triggered by IFN‐β/γ. As shown in Figure S4A,B (Supporting Information), cell apoptosis or cleavage of caspases‐3 were not observed following IFN‐β/γ treatment. Furthermore, inhibition of cell death by a pan‐caspase inhibitor (Z‐VAD‐FMK) had no marked effects on the transcription of *Cxcl10* and *Ifit1* genes induced by IFN‐β/γ (Figure S4C, Supporting Information). These observations eliminate the possibility that IFN‐β and IFN‐γ trigger mtDNA release by inducing cell death. We then assessed the effects of IFN‐β‐ and IFN‐γ treatment on mitochondrial function, indicated by the production of reactive oxygen species (ROS). The results showed that treatment with IFN‐β/γ enhanced the production of mtROS (Figure S4D, Supporting Information), suggesting that IFN‐β/γ stimulation triggers mitochondrial dysfunction. Additionally, we found that the protein level of VDAC1 was elevated following IFN‐β stimulation, accompanied by an increase of VDAC1 oligomerization (Figure S4E,F, Supporting Information). Consistently, treatment with VBIT‐4, a highly potent inhibitor of VDAC1 oligomerization, resulted in the suppression of IFN‐β/γ‐induced transcription of *Cxcl10*, *Ifit1* but not *Irf1* genes (Figure S4G, Supporting Information). These results suggest that IFN‐β/γ induces expression and oligomerization of VDAC1, as well as mitochondrial dysfunction, which contributes to the release of mtDNA.

### 2.4 IFNs Induce JAK1‐mediated Tyrosine Phosphorylation and Activation of cGAS

**Figure 5:**
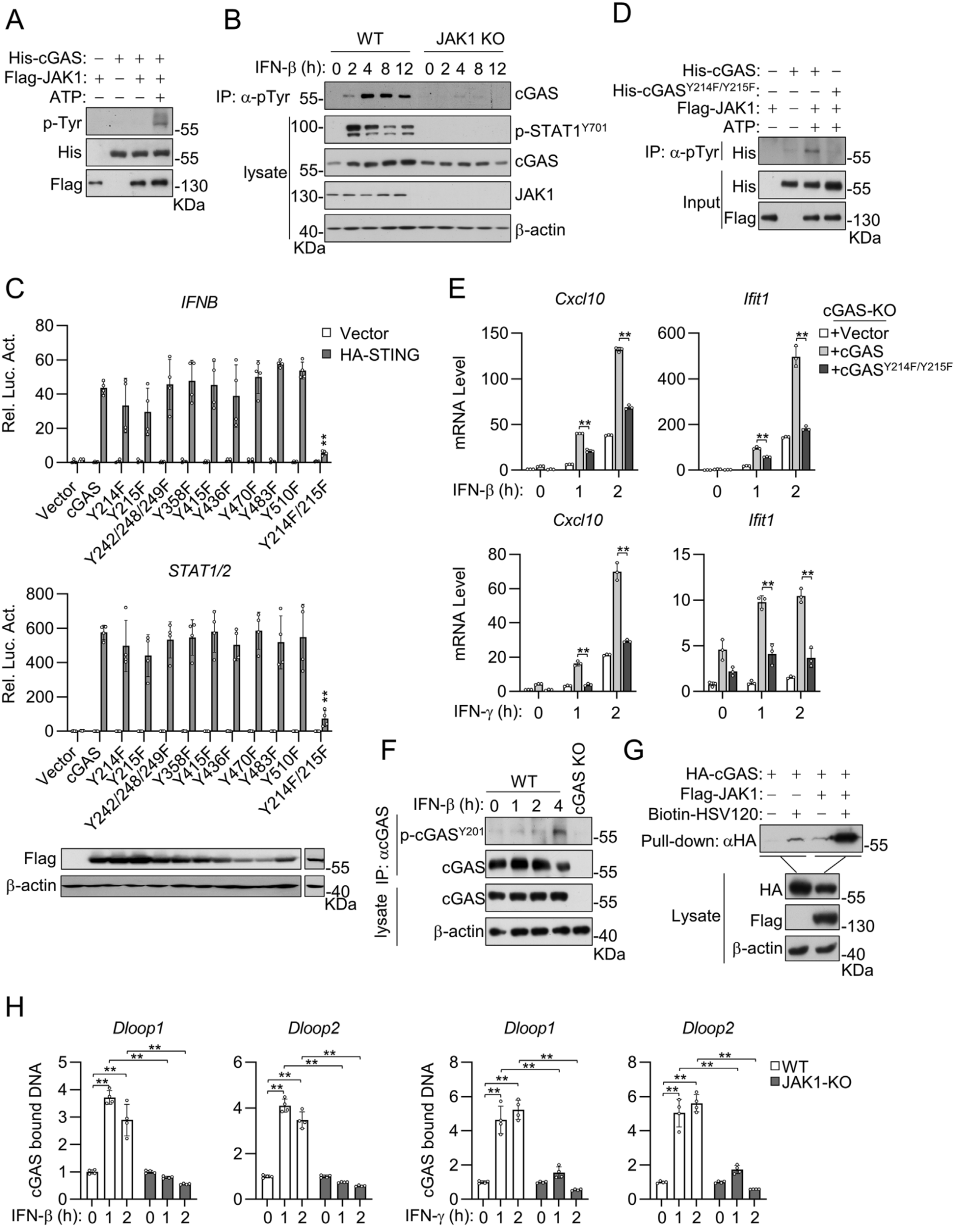
IFNs induce JAK1‐mediated cGAS tyrosine phosphorylation and activation. A) JAK1 directly phosphorylates cGAS in vitro kinase assay. *Escherichia coli*‐derived His‐cGAS and HEK293‐dervived Flag‐JAK1 was incubated in the presence or absence of ATP. Tyrosine phosphorylation of cGAS was examined by immunoblotting analysis with a phosphor‐tyrosine specific antibody. B) Knockout of JAK1 inhibits IFN‐β‐induced tyrosine phosphorylation of cGAS. Co‐immunoprecipitation and immunoblot analysis of tyrosine phosphorylation of cGAS were performed in WT and JAK1‐deficient MLFs stimulated with IFN‐β (20 ng mL^−1^) for the indicated times. C) HEK293 cells were transfected with the indicated reporter (*IFNB* promoter and STAT1/2) and expression (HA‐STING, Flag‐cGAS or its mutants) plasmids for 24 h before luciferase assays were performed. D) Mutation of Y214/215 of cGAS inhibits its tyrosine phosphorylation by JAK1. *Escherichia coli*‐derived His‐cGAS or His‐cGAS^Y214F/Y215F^ and HEK293‐derived Flag‐JAK1 were incubated in the presence or absence of ATP. The mixture was pulled down with anti‐pTyr and analyzed by immunoblotting with the indicated antibodies. E) Effects of reconstitution of cGAS and cGAS^Y214F/Y215F^ on IFN‐β‐ and IFN‐γ‐triggered transcription of downstream genes in cGAS‐deficient MLFs. The cells were treated with IFN‐β (20 ng mL^−1^) or IFN‐γ (20 ng mL^−1^) for the indicated times before qPCR analyses for the indicated genes were performed. F) IFN‐β induces Y201 phosphorylation of mouse cGAS. WT and cGAS‐deficient MLFs were stimulated with IFN‐β (20 ng mL^−1^) for the indicated times. Cell lysates were collected and immunoprecipitated with cGAS antibody before being analyzed by immunoblotting with the indicated antibodies. G) JAK1 enhances the DNA‐binding ability of cGAS. HEK293 cells were transfected with the indicated plasmids for 24 h. The cell extracts were incubated with biotinylated HSV120 and streptavidin agarose for 3 h. The bound proteins were analyzed by immunoblots with the indicated antibodies. H) Knockout of JAK1 inhibited cGAS binding to mtDNA. WT and JAK1‐knockout MLFs were unstimulated or stimulated with IFN‐β (20 ng mL^−1^) or IFN‐γ (20 ng mL^−1^) for the indicated times. Cell lysates were collected and immunoprecipitated with cGAS antibody, followed by qPCR analysis of extracted DNA to detect cGAS‐bound mtDNA abundance. The fold enrichment of mtDNA in cGAS immunoprcipitates in cells treated with IFN‐β or IFN‐γ was calculated as compared with that in un‐stimulated cells. Data are shown as mean ± SD (C, H *n* = 4; E, *n* = 3), and statistical significance was determined by one‐way ANOVA (E) or unpaired two‐tailed Student‘s *t‐*test (C, H). **p < 0.01.

Since JAK1 is an essential tyrosine kinase activated by IFNs,^[^ ^28^ ^]^ we investigated whether cGAS is phosphorylated by JAK1. In vitro kinase assays indicated that JAK1 directly phosphorylated cGAS (**Figure** **5**A). Knockout of JAK1 inhibited tyrosine phosphorylation of cGAS after IFN‐β stimulation (Figure 5B). These results suggest that JAK1 mediates the tyrosine phosphorylation of cGAS after IFN‐β stimulation.

We next mapped which tyrosine residues of cGAS are targeted by JAK1. We mutated all of the tyrosine residues in human cGAS to phenylalanine individually or in different combinations. These cGAS mutants were co‐transfected with STING to examine their abilities to activate the IFN‐β promoter or STAT1/2 luciferase reporter in HEK293 cells. The results indicated that mutation of any single tyrosine residue of cGAS had no marked effects on its ability to activate the IFN‐β promoter or STAT1/2 reporter, whereas simultaneous mutation of Y214 and Y215 to phenylalanine (Y214F/Y215F) dramatically inhibited its activity (Figure 5C). In vitro kinase assays indicated that JAK1 phosphorylated wild‐type cGAS but not cGAS^Y214F/Y215F^ (Figure 5D). Immunoblotting using an antibody specifically targeting Y201‐phosphorylated mouse cGAS (equivalent to Y215 of human cGAS) confirmed cGAS phosphorylation at this site following IFN‐β treatment (Figure 5F). Reconstitution with cGAS but not cGAS^Y214F/Y215F^ rescued transcription of the downstream *Cxcl10* and *Ifit1* genes induced by IFN‐β and IFN‐γ in cGAS‐deficient MLFs (Figure 5E). Additionally, DNA pull‐down assays indicated that JAK1 enhanced the ability of cGAS to bind DNA (Figure 5G). In contrast, knockout of JAK1 markedly inhibited cGAS binding to mtDNA after IFN‐β and IFN‐γ treatment (Figure 5H). These results suggest that tyrosine phosphorylation of cGAS^Y214/Y215^ by JAK1 promotes its ability to bind DNA, which is consistent with a previous finding that efficient activation of cGAS requires its phosphorylation at Y214/Y215 to provide a priming signal.^[^ ^29^ ^]^

### 2.5 JAK1‐cGAS‐STING‐IRF3 Axis is Important for Host Antiviral Response

**Figure 6:**
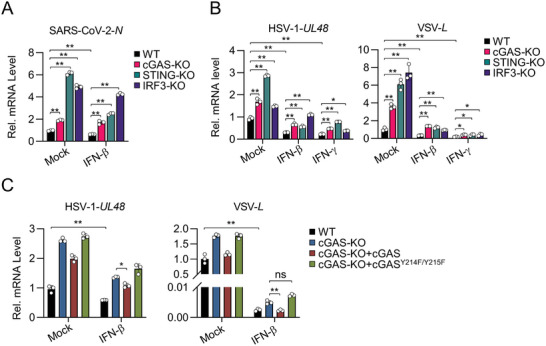
The JAK1‐cGAS‐STING‐IRF3 axis is important for host antiviral response. A) WT, cGAS‐, STING‐ or IRF3‐deficient MLFs transfected with human ACE2 were unpretreated or pretreated with mIFN‐β (20 ng mL^−1^) for 1 h and then infected with SARS‐CoV‐2 (MOI = 1) for 24 h. qPCR of the indicated viral RNAs was performed. The values were normalized to that of unpretreated WT cells. B) WT, cGAS‐, STING‐ or IRF3‐deficient MLFs were unpretreated or pretreated with mIFN‐β (20 ng mL^−1^) and mIFN‐γ (20 ng mL^−1^) for 1 h and then infected with HSV‐1 for 24 h or VSV for 12 h. qPCR of the indicated viral RNAs was performed. The values were normalized to that of unpretreated WT cells. C) WT and cGAS‐deficient MLFs reconstituted with empty vector, cGAS or cGAS^Y214F/Y215F^ mutant were unpretreated or pretreated with IFN‐β (20 ng mL^−1^) for 1 h and then infected with HSV‐1 or VSV for 12 h before qPCR analysis of the indicated viral RNAs was performed. The values were normalized to that of the unpretreated WT cells. Data are shown as mean ± SD (n = 3), and statistical significance was determined by two‐way ANOVA. *p < 0.05; **p < 0.01. ns, not significant.

Previous studies have established the roles of the cGAS‐STING pathway in the host response to DNA viruses.^[^ ^3^, ^4^ ^]^ Interestingly, recent studies also imply that cGAS and STING regulate the host response to certain RNA viruses by sensing mtDNA released into the cytosol upon infection,^[^ ^3^, ^11^ ^]^ STING‐dependent translational inhibition,^[^ ^30^ ^]^ and membrane fusion‐triggered IFN production.^[^ ^31^ ^]^ We next explored whether IFN‐JAK1‐mediated activation of cGAS‐STING‐IRF3 is a common antiviral mechanism for different viruses. To this end, we first examined the antiviral roles of cGAS, STING, and IRF3 in responses to different viruses, including the double‐stranded (ds) DNA virus HSV‐1, the positive‐sense (+ss) RNA virus SARS‐CoV‐2 and the negative sense single‐stranded (‐ssRNA) virus vesicular stomatitis virus (VSV). We found that knockout of cGAS, STING, and IRF3 promoted replication of all tested viruses as indicated by increased viral mRNA in the infected cells (**Figure** **6**A,B). Furthermore, the inhibitory effects of IFN‐β or IFN‐γ on replication of HSV‐1, SARS‐CoV‐2, and VSV were reversed in cGAS‐, STING‐ and IRF3‐deficient cells (Figure 6A,B). To further verify whether IFN‐activated antiviral activity is dependent on JAK1‐mediated cGAS phosphorylation, we reconstituted cGAS and cGAS^Y214F/Y215F^ in cGAS‐deficient cells and evaluated the antiviral effects of these cell lines. The results showed that reconstitution of cGAS but not cGAS^Y214F/Y215F^ rescued the antiviral effects of IFN‐β in cGAS‐deficient cells against HSV‐1 and VSV infection (Figure 6C), suggesting that JAK1‐mediated phosphorylation of cGAS is important for IFN‐mediated antiviral activity.

### 2.6 The cGAS‐IRF3 Axis Plays an Important Role in IFN‐triggered Antitumor Activity

**Figure 7:**
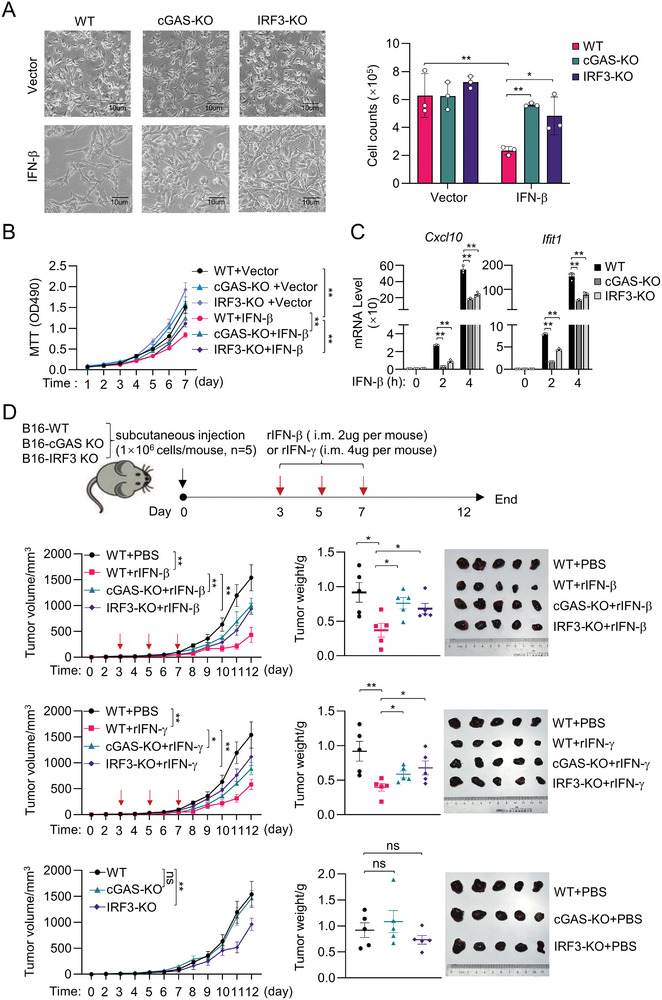
Knockout of cGAS and IRF3 impairs IFN‐β‐mediated antitumor effects. A&B) Knockout of cGAS and IRF3 inhibits IFN‐β‐mediated inhibition of proliferation in B16‐F10 cells. The WT‐, cGAS‐KO‐ or IRF3‐KO‐B16‐F10 cells were 1:1 mixed with either the vector‐expressing‐ or IFN‐β‐expressing‐ B16‐F10 cells as indicated and cultured for 2 days. The mixed cell cultures were photographed (A, left) and the cell numbers were counted (A, right). The cell viability was measured by MTT assay (B) at the indicated time points. C) Knockout of cGAS and IRF3 inhibits IFN‐β‐induced transcription of *Cxcl10* and *Ifit1* in B16‐F10 cells. qPCR analysis of mRNA abundance of the indicated genes in WT‐, cGAS‐KO‐, and IRF3‐KO‐B16‐F10 cells stimulated with IFN‐β (20 ng mL^−1^) or IFN‐γ (20 ng mL^−1^) for the indicated times. D) Knockout of cGAS or IRF3 impairs the inhibitory effects of IFN‐β and IFN‐γ on tumor growth. BALB/C‐Nu mice were allografted with WT, cGAS‐, or IRF3‐deficiency B16F10 cells (1 × 10^6^ 50 µL, s.c.). Intratumor injection of PBS, recombinant mIFN‐β (2 µg per mouse), or mIFN‐γ (4 µg per mouse) was given on days 3, 5, or 7 after the graft. In this experiment, the mIFN‐β and mIFN‐γ‐treated groups shared the same control PBS group. Tumor volume was monitored daily and calculated as LW^2^/2 (L represents the longest diameter of the tumor, and *W* represents the maximum horizontal diameter in the vertical direction). Tumors were weighed and photographed at the endpoint (day 12). Data are shown as mean ± SD (A, C, *n* = 3) or ± SEM (B, D, *n* = 5), statistical significance was determined by one‐way ANOVA (A, C) or two‐way ANOVA (B, D). *p < 0.05; **p < 0.01.

In addition to antiviral activities, previous studies have demonstrated that type I IFNs have antitumor activities. Next, we utilized a melanoma xenograft model to investigate whether the cGAS‐STING‐IRF3 axis is involved in IFN‐β‐triggered antitumor activity. As previously reported,^[^ ^32^ ^]^ IFN‐β treatment significantly inhibited the proliferation of the B16‐F10 melanoma cells, whereas knockout of cGAS and IRF3 reversed the inhibitory effects of IFN‐β (**Figure** **7**A,B). Moreover, IFN‐β‐induced transcription of *Cxcl10* and *Ifit1* genes was inhibited in cGAS‐ and IRF3‐deficient B16‐F10 cells in comparison to control cells (Figure 7C). In a xenograft tumor model, treatment of IFN‐β or IFN‐γ inhibited tumor growth of wild‐type but not cGAS‐ or IRF3‐deficient B16‐F10 cells (Figure 7D). These results suggest that the cGAS‐IRF3 axis plays an important role in IFN‐β‐ and IFN‐γ‐triggered antitumor immunity.

## 3 Discussion

Microbial DNA, dis‐located cellular DNA and mtDNA are sensed by cGAS, which activates the STING‐TBK1‐IRF3 axis to induce expression of host defense genes including type I IFNs and proinflammatory cytokines.^[^ ^33^ ^]^ Type I IFNs are secreted cytokines that bind to IFNAR and activate JAK‐STAT pathways in autocrine and paracrine processes, leading to amplification of IFNs as well as expression of various effector genes in infected and uninfected cells.^[^ ^13a^ ^]^ Recently, IRF3 has been reported to play unconventional functions including inhibiting nuclear translocation of NF‐κB and activating RB signaling.^[^ ^34^ ^]^ In this study, we found that IRF3 is directly activated following type I and II IFN stimulation, and this is important for the antiviral and antitumor activities of IFNs.

There are several lines of evidence suggesting that IFNs activate cGAS‐STING‐TBK1‐IRF3 axis to amplify the expression of a set of innate immune effector genes. Stimulation of IFN‐β or IFN‐γ induced phosphorylation of STING^S365^, TBK1^S172^, and IRF3^S379^, which are hallmarks of their activation respectively. Knockout of cGAS, STING but not VISA inhibited either IFN‐β or IFN‐γ‐induced phosphorylation of TBK1^S172^ and IRF3^S379^. Consistently, knockout of cGAS, STING, TBK1, or IRF3 inhibited IFN‐β‐ and IFN‐γ‐triggered transcription of a set of ISRE‐containing genes. Interestingly, knockout of cGAS, STING, TBK1, or IRF3 had no marked effects on transcription of *Irf1* gene, which is controlled by STAT1/2‐binding GAS‐containing promoter. Consistently, wild‐type IRF3 but not its transcriptional inactive mutants rescued IFN‐triggered induction of these downstream genes in IRF3‐deficient cells. These results suggest that the cGAS‐STING‐TBK1‐IRF3 axis is activated by both type I and II IFNs, which drives the transcription of a set of ISRE‐containing genes after IFN stimulation, providing an alternative mechanism for the induction of ISRE‐containing ISGs.

In addition to the direct induction of ISRE‐containing genes upon IFN stimulation, our results suggest that the cGAS‐STING‐TBK1‐IRF3 axis also regulates the basal levels of STAT1 and STAT2. Knockout of cGAS, STING, TBK1, or IRF3 caused down‐regulation of basal STAT1/2 but not STAT3 in unstimulated cells. Interestingly, although the absolute levels of phosphorylated STAT1/2 following IFN stimulation were minimally decreased in cGAS‐, STING‐ or IRF3‐deficient cells, the ratios of phosphorylated to total STAT1/2 following IFN stimulation in these knockout cells were even higher than wild‐type control cells, suggesting that the cGAS‐STING‐IRF3 axis is not required for STAT1/2 phosphorylation and activation per se. Knockout of cGAS, STING, TBK1 or IRF3 also caused down‐regulation of basal mRNA levels of STAT1/2 but not STAT3. The simplest explanation for these results is that the basal activity of the cGAS‐STING‐TBK1‐IRF3 axis is important for inducing STAT1/2 transcription as well as maintaining them at proper levels in un‐stimulated cells. Therefore, the cGAS‐STING‐TBK1‐IRF3 axis is an important arm of the IFN‐triggered induction of downstream effector genes not only by direct induction of downstream genes but also indirectly via inducing STAT1/2. How cGAS maintains basal activity is not clear. Interestingly, a recent study suggests that extrachromosomal circular DNA (cccDNA) activates STING‐dependent innate immune response in unstimulated cells.^[^ ^35^ ^]^

Our results suggest that IFN activates the cGAS‐STING‐TBK1‐IRF3 axis by two processes. First, IFN‐β and IFN‐γ stimulation caused mitochondrial dysfunction and release of mtDNA in the cytosol. Depletion of mtDNA by EtBr inhibited IFN‐β‐induced cGAS‐STING activation and transcription of downstream effector genes. These results suggest upon IFN stimulation, mtDNA released into the cytosol is sensed by cGAS. Second, our results suggest that IFN stimulation triggers JAK1‐mediated phosphorylation of cGAS. Knockout of JAK1 inhibited IFN‐β‐triggered tyrosine phosphorylation of cGAS in cells, whereas recombinant JAK1 phosphorylated cGAS directly in in vitro kinase assays. Mutagenesis indicated that JAK1 phosphorylated human cGAS at Y214/Y215. Reconstitution of wild‐type cGAS but not cGAS^Y214F/Y215F^ rescued IFN‐β or IFN‐γ‐triggered induction of *Cxcl10* and *Ifit1* genes. In addition, JAK1‐mediated phosphorylation of cGAS promoted its mtDNA‐binding activity. These results suggest that IFN stimulation causes JAK1‐mediated tyrosine phosphorylation of cGAS at Y214/Y215, which is important for its mtDNA binding and activation. Although in vitro experiments demonstrate that cGAS is able to directly bind DNA without additional cofactors,^[^ ^25^ ^]^ in a cell with few DNA ligands, optimal activation of cGAS may require other additional regulatory mechanisms such as the formation of liquid‐like droplets,^[^ ^36^ ^]^ increased level of cytosolic ion ^[^ ^37^ ^]^ and post‐translation modifications.^[^ ^4a^ ^]^ Our results suggest that priming of cGAS by tyrosine phosphorylation and mtDNA exposure in the cytosol are two processes required for efficient activation of the cGAS‐STING‐IRF3 axis following IFN stimulation.

The cGAS‐STING pathway is originally defined as a DNA recognition signal that limits DNA virus infection in host cells by activating innate immune responses. Several subsequent studies have identified cGAS as an important player in inhibiting +ssRNA viral infections such as West Nile virus (WNV),^[^ ^38^ ^]^ dengue virus (DENV),^[^ ^11b,c^ ^]^ and SARS‐CoV2.^[^ ^39^ ^]^ In the life cycles of these viruses, the massive rearrangements of intracellular membranes may trigger the mislocalization of cellular DNA that serves as a substrate to activate the cGAS‐STING pathway. Another study suggests that the influenza virus M2 protein triggers the translocation of mtDNA into the cytosol to stimulate cGAS‐ and DDX41‐dependent innate immune responses.^[^ ^11a^ ^]^ Previously it has been assumed that cytosolic PRR signaling is restricted to infected cells. Our findings expand the possibility of the cGAS‐STING axis as a broad‐spectrum antiviral arm in infected and un‐infected cells as well as a mediator of antitumor activity triggered by type I and II IFNs. Being parallel with the JAK‐STAT pathway, the JAK1‐cGAS‐STING‐IRF3 axis may play a complementary role when the JAK‐STAT pathway is hijacked by the virus.^[^ ^40^ ^]^ Taken together, our findings provide additional insights into the complicated mechanisms of host defense against pathogens and tumorigenesis.

## 4 Experimental Section

### Mice

*Irf3^−/−^ *mice were kindly provided by Dr. Xinwen Chen (Wuhan Institute of Virology). The genotyping of *Irf3^−/−^* mice was confirmed by PCR with the following primers: WT: GAACCTCGGAGTTATCCCGAAGG (forward) and GTTTGAGTTATCCCTGCACTTGGG (reverse); KO: GAACCTCGGAGTTATCCCGAAGG (forward) and TCGTGCTTTACGCTATCGCCGCTCCCGATT (reverse). All mice were housed in groups of 5 mice per cage on a 12‐h light/dark cycle in a temperature‐controlled specific pathogen‐free (SPF) room (23–25 °C, relative humidity of 40%–70%) with free access to water and food. At the experimental endpoint, animals were sacrificed by cervical dislocation after isoflurane anesthesia. Viral infection experiments were performed in the ABSL‐2 facility at the Wuhan Institute of Virology. The experimental protocol has adhered to the International Guiding Principles for Biomedical Involving Animals. The protocol for animal experiments was approved by the Institutional Animal Care and Use Committee of the Wuhan Institute of Virology (approval number WIVA31202107).

### Cells

HEK293 and pHFF cells were purchased from ATCC and cultured in DMEM (Hyclone) supplemented with 10% (vol/vol) FBS (Gibco), 100 U/ml penicillin, and 100 µg mL^−1^ streptomycin (Gibco). pMLFs were prepared from age‐ and sex‐matched wild‐type and *Irf3* knockout mice and cultured in DMEM/F‐12 medium (Hyclone) supplemented with 10% (vol/vol) FBS (Gibco), 100 U mL^−1^ penicillin and 100 µg mL^−1^ streptomycin (Gibco). Immortalized MLF (iMLF) was generated as previously described.^[^ ^41^ ^]^ Bone marrow cells were isolated from the tibia and femur. For the preparation of BMDMs, the bone marrow cells were cultured in RPMI 1640 medium supplemented with 10% FBS and 10% M‐CSF containing supernatant from L929 cells for 4 days. All cell lines were cultured at 37 °C and 5% CO_2_.

### Reagents, Viruses and Antibodies

Recombinant mouse IFN‐β (Biolegend, 581 302), human IFN‐β (Peprotech, #300‐02BC), Recombinant mouse IFN‐α (Biolegend, 752 802), mouse IFN‐γ (Biolegend, 575 302), human IFN‐γ (Peprotech, #300‐02), mouse IL‐1β (Peprotech, #211‐11B), mouse TNF‐α (Peprotech, #315‐01A), puromycin (Life Science, 58‐58‐2), Cycloheximide (MCE, HY‐12320), blasticidin (Thermo, R21001), polybrene transfection reagent (Specialty Media, TR‐1003‐G), protein G sephorase (Cytiva, 17 061 805), CDDP (MCE, HY‐17394), VBIT‐4 (TargetMol, 2086257‐77‐2), Z‐VAD‐FMK (MCE, HY‐16658B ), digitonin (Sigma, D5628), HT‐DNA (Sigma, 31 149), ethidium bromide (Aladdin, E119045), RNAiso Plus (Takara, 9109) and type II collagenase (Invitrogen, 17 101 015) were purchased from the indicated companies. Biotin‐labeled HSV120 was synthesized by Sangon Biotech, and the sequences are shown in Table S1 (Supporting Information). VSV and HSV‐1 (KOS strain) were purchased from the China Center for Type Culture Collection. The SARS‐CoV‐2 original strain (IVCAS 6.7512) was obtained from the National Virus Resource Center and propagated in Vero E6 cells. All experiments involving viruses were conducted in respective Biosafety Level 2 or 3 laboratories. Information on the commercially available antibodies used in this study is provided in Table S2 (Supporting Information).

### Constructs

STAT1/2 and IFN‐β promoter luciferase reporter plasmids were purchased from Qiagen. Mammalian expression plasmids for Flag‐tagged IRF3 and its mutants were constructed into the pMSCV vector. Mammalian expression plasmids for Flag‐tagged cGAS were constructed into the pLOV‐CMV‐eGFP‐2A‐EF1a‐BSD or pRK vector. An expression plasmid for mIFN‐β was constructed into the pLOV‐CMV‐eGFP‐2A‐EF1a‐Puro vector. HA‐tagged cGAS, HA‐tagged STING, and Flag‐tagged JAK1 were constructed into the pRK vector. The mutants of IRF3 and cGAS were constructed by site‐directed mutagenesis. The information on primers for site‐directed mutagenesis is shown in Table S3 (Supporting Information). All plasmids were constructed by standard molecular biology techniques.

### CRISPR‐Cas9 Knockout

Double‐stranded oligonucleotides corresponding to the target sequences were cloned to the lenti‐CRISPR‐V2 vector. HEK293 cells were co‐transfected with lenti‐CRISPR plasmid (10 µg) and packaging plasmids (pSPAX2 7.5 µg and pMD2.G 5 µg). Twelve hours after transfection, the medium was replaced. Two days later, the viruses were harvested and filtered (0.22 µm filter), then added into MLFs or RAW264.7 cells in the presence of polybrene (8 µg mL^−1^). Twenty‐four hours after infection, the infected cells were selected with puromycin (0.5 µg mL^−1^) or blasticidin (10 µg mL^−1^) for at least 5 days. The information on gRNA sequences is shown in Table S4 (Supporting Information).

### Transfection and Reporter Assay

HEK293 cells were transfected by standard calcium phosphate precipitation method. MLFs were transfected by Lipofectamine 2000. For reporter assays, HEK293 cells were seeded on 48‐well plates and transfected by the standard calcium phosphate precipitation method. Control plasmid was added to ensure that each transfection receives the same amount of total DNA. To normalize transfection efficiency, 10 ng of pGL‐TK reporter plasmid was added to each transfection. Luciferase assays were performed using a dual‐specific luciferase assay kit (Promega).

### Small Interfering RNA (siRNA)

siRNAs were designed and synthesized by RiboBio Co., Ltd. The sequences of IRF3 siRNA: 5′‐GAUCUGAUUACCUUCACGGAAdTdT‐3′ (sense), 3′‐dTdTCUAGACUAAUGGAAGUGCCUU‐5′ (antisense). pHFF cells were seeded in 12‐well plates at a density of 2 × 10^5^/well. The cells were transfected with 20 nM siRNA using GenMute siRNA transfection kit (SignaGen Laboratories) according to the manufacturer's protocol. The experiments were performed 36 h post transfection.

### RNA Extraction and qPCR

Total RNA was extracted using the TRizol reagent. RNAs were reverse‐transcribed to cDNA for qPCR analysis to measure mRNA levels of the indicated genes, which were normalized to *Gapdh* mRNA. Gene‐specific primer sequences were described in Table S5 (Supporting Information).

### Immunoblots

Cells were lysed in lysis buffer (20 mM Tris‐HCl, pH 7.4, 150 mM NaCl, 1 mM EDTA, 1% NP‐40) supplemented with complete protease inhibitor mixture (Targetmol) by incubation on ice for 15 min, and cleared of insoluble materials by centrifugation. The lysates were fractionated by SDS‐PAGE and transferred to a nitrocellulose filter membrane (Millipore). The antibodies used for immunoblots are listed in Table S2 (Supporting Information).

### Co‐Immunoprecipitation Assay

Cells were lysed in 1 mL lysis buffer (20 mM Tris‐HCl, pH 7.4, 150 mM NaCl, 1 mM EDTA, 1% NP‐40) supplemented with protease and phosphatase inhibitors. The cell lysate was incubated with 0.5 µg of the indicated antibody and 40 µL of a 50% slurry of Protein G‐Sepharose (GE Healthcare) at 4 °C for 4 h. The Sepharose beads were then washed three times with 1 mL of lysis buffer containing 0.1 M NaCl. The beads were resuspended with 60 µL 2×SDS loading buffer and boiled for 10 min at 95 °C. The associated proteins were analyzed by SDS‐PAGE immunoblots as described above.

### Measurement of mtDNA

Subcellular fractionation and mtDNA quantification were performed similarly as described.^[^ ^3b^ ^]^ Cells (5 × 10^5^) were lysed in 100 µL digitonin buffer (150 mM NaCl, 50 mM HEPES pH 7.4, 50 µg mL digitonin, and full protease and phosphatase inhibitors) and incubated on a rotator at 4 °C for 10 min. The lysis was divided into two aliquots with one aliquot used to quantitate cellular genomic DNA. Another aliquot was centrifuged at 2 000 g for 10 min at 4 °C. The supernatant was transferred to a fresh tube and centrifuged at 20 000 g for 20 min at 4 °C, and this was repeated 3 times. The yield cytosolic fraction was split into two tubes (one for mtDNA extraction and one for immunoblotting analysis). DNA was isolated using QIAQuick Nucleotide Removal Column (QIAGEN). qPCR was performed using nuclear DNA primers (*Tert*) and mtDNA primers (*D‐loop* 1–2). The Ct values for nuclear DNA abundance of whole‐cell extracts served as normalization controls for the mtDNA values obtained from the cytosolic fractions.

### mtDNA‐Depleted Cells

Depletion of mtDNA was performed in a culture medium containing 100 ng mL^−1^ ethidium bromide, 100 mg mL^−1^ sodium pyruvate, and 50 µg mL^−1^ uridine for 2–3 weeks as previously demonstrated.^[^ ^42^ ^]^ The depletion efficiency was evaluated by qPCR analysis of mitochondrial genes.

### cGAS‐mtDNA Binding Assay

MLFs were seeded in 10 cm dishes and stimulated with IFN‐β for the indicated times. Cells were fixed with 1% formaldehyde for 10 min. Fixation was stopped by the addition of glycine to a final concentration of 125 mM. The cells were washed with cold PBS 3 times and then collected by centrifugation. The cell pellets were lysed in lysis buffer (50 mM Tris‐HCl, pH 8.0, 5 mM EDTA, 0.5% SDS) for 15 min and sonicated for 30 with 30 s intervals for 10 cycles. An aliquot of the lysate was used as input. The rest of the cell lysate was incubated with mouse cGAS antibody overnight at 4 °C and then incubated with 40 µL of a 50% slurry of protein G‐depharose (GE Healthcare) for 4 h at 4 °C. The beads were then sequentially washed with washing buffer I (20 mM Tris‐HCl, pH 8.0, 150 mM NaCl, 2 mM EDTA, 1% Triton‐100, 0.1% SDS), washing buffer II (20 mM Tris‐HCl, pH 8.0, 500 mM NaCl, 2 mM EDTA, 1% Triton‐100, 0.1% SDS) and TE buffer (20 mM Tris‐HCl, pH 8.0, 2 mM EDTA) before incubated with elution buffer (0.1 M NaHCO3, 1% SDS, 100×proteinase K) for 15 min at room temperature. The eluted samples were incubated with 0.5 M NaCl containing proteinase K (200 µg mL^−1^) overnight at 65 °C for de‐crosslinking. The precipitated DNA and input DNA were extracted from the elute and the input respectively with phenol‐chloroform and analyzed by qPCR. The fold enrichment of mtDNA in cGAS immunoprcipitates in cells treated with IFNs compared to mock‐transfected cells was calculated.

### Measurements of ROS

Mitochondrial ROS levels were assessed by mitoSOX (Invitrogen, M36008). MLFs (1 × 10^5^) were seeded in 96 well plates and stimulated with IFN‐β, IFN‐γ, or FCCP for the indicated times. Then the cells were stained with mitoSOX (5 µM) for 20 min at 37 °C with 5% CO_2._ The fluorescence intensity of the cells was quantified using High Content Imaging. FCCP‐treated cells were presented as a positive control.

### Apoptosis Assay

Apoptosis assays were conducted using an AnnexinV‐FITC Apoptosis Detection Kit I (BD, 556 547) according to the manufacturer's recommended protocol. Briefly, treated cells (1 × 10^5^) were collected, washed twice with ice‐cold PBS, and resuspended in 500 µL of binding buffer containing Annexin V‐FITC (5 µL) and propidium iodide (5 µL). The treated cells were placed in the dark at room temperature for 30 min, after which flow cytometry analysis was performed.

### Purification of Recombinant Proteins

For cGAS and mutant purification, the plasmids encoding his‐cGAS and his‐cGAS^Y214F/Y215F^ were transformed into *E. coli* BL21 (DE3). Expression of the recombinant proteins was induced with 0.1 mM IPTG at 16 °C for 6 h. Ni^2+^‐NTA‐agarose was used for the purification of His‐tagged cGAS protein. For JAK1 purification, a mammal expression plasmid for Flag‐JAK1 was transfected into HEK293 cells. The cells were lysed 18 h after transfection. Flag antibody‐conjugated beads were then used for immunoprecipitation for 4 h at 4 °C. The beads were washed three times with lysis buffer. The Flag‐tagged JAK1 was eluted with 3xFlag peptide in 25 mM Tris‐HCl, pH 8.0.

### In Vitro Kinase Assay

Recombinant cGAS proteins and Flag‐JAK1 were incubated at 30 °C for 1 h in buffer (25 mM HEPES, pH 7.3, 10 mM MgCl_2_, 0.5 mM Na_3_VO_4_, 2 mM DTT, 0.5 mM ATP). After the reaction, 10 µL 6×SDS loading buffer was added. Reactions were detected by SDS‐PAGE and immunoblotting analysis.

### Tumor Models

Cells in the exponential growth phase were harvested by trypsinization and washed twice in PBS before injection. For the s.c. injections, B16‐F10 cells (1×10^6^ in 50 µL of PBS) were injected into the abdominal s.c. space of six‐week‐old female BALB/c‐Nu mice were sourced from the Animal Center of Wuhan Institute of Virology. Tumor growth at the skin was monitored by measurement of the two maximum perpendicular tumor diameters, and the tumor volumes were calculated as LW^2^/2 (L represents the longest diameter of the tumor, and W represents the maximum horizontal diameter in the vertical direction).^[^ ^43^ ^]^ On day 12 postimplantation, mice were sacrificed and tumors were harvested for experiments.

### MTT Assay

Cells were plated in 96‐well plates at a density of 1000 cells/well, and the cell viability was measured by the 3‐(4,5‐dimethylthiazol‐2‐yl)−2,5‐diphenyltetrazolium bromide (MTT) assay at every day.

### Statistics and Reproducibility

Statistical analysis was performed with Prism Version 8.0 (GraphPad). Statistical significance was analyzed by one‐way ANOVA analysis, followed by Dunnett's test. Two‐tailed unpaired (Student) t‐test was performed if only two conditions were compared and the F test was performed to confirm that 2 populations have the same variances. The Shapiro‐Wilk normality test was performed to confirm the normal distribution of all datasets. All data are representative of at least two independent experiments with similar results.

## Conflict of Interest

The authors declare no conflict of interest.

## Supporting information

Supporting Information

## Data Availability

The data that support the findings of this study are available from the corresponding author upon reasonable request.
