# Supplementary material for: Activation of the cGAS‐STING‐IRF3 Axis by Type I and II Interferons Contributes to Host Defense
Source: Adv Sci (Weinh). 2024 Jul 14;11(35):2308890. doi: 10.1002/advs.202308890 (PMC11425201; doi:10.1002/advs.202308890)

Supporting Information

Activation of the JAK1-cGAS-STING-IRF3 axis by type I and II interferons contributes to host defense

Zhen Tong[1,2], Jia-Peng Zou[1,2], Su-Yun Wang[1], Wei-Wei Luo[1,2,3]*, Yan-Yi Wang[1,2,]*

This PDF file includes:

Figures S1 to S5

Tables S1 to S5

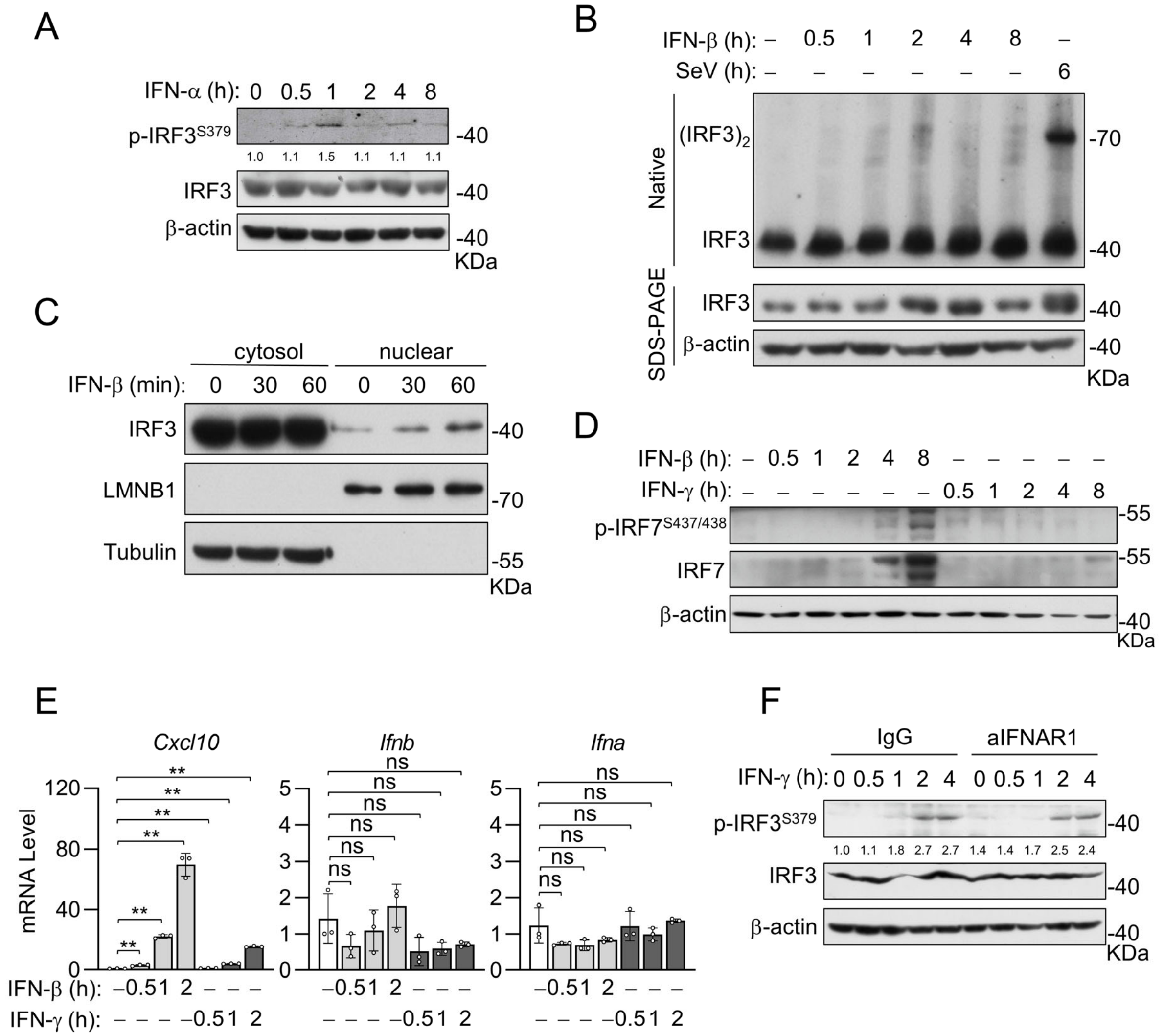

Figure S1. IFN induces activation of IRF3

(A) IFN-α induces IRF3 phosphorylation. MLFs were stimulated with IFN-α (40 ng/mL) for the indicated times before immunoblot analysis of the indicated proteins.
(B) IFN-β induces IRF3 dimerization. Cells were stimulated with IFN-β (20 ng/mL) or infected with SeV for the indicated times before immunoblot analysis of the indicated proteins by SDS-PAGE. IRF3 dimerization was analyzed by Native-PAGE.
(C) Cell fractionation analysis of RAW264.7 stimulated with IFN-β (20 ng/mL) for the indicated times. The nuclear (Nuc.) and cytoplasmic (Cyto.) extracts were analyzed by immunoblotting with the indicated antibodies.
(D) Effects of IFN-β and IFN-γ treatment on expression and phosphorylation of IRF7. Immunoblot analysis of the indicated proteins in MLFs stimulated with IFN-β (20 ng/mL) or IFN-γ (20 ng/mL).

(E) IFN-β or IFN-γ did not induce the primary transcription of *Ifnb1* and *Ifna*. MLFs were stimulated with IFN-β (20 ng/mL) or IFN-γ (20 ng/mL) for the indicated times. The mRNA abundance of the indicated genes was measured by qPCR. Data are shown as mean ± SD (n=3), statistical significance was determined by unpaired two-tailed Student's *t* test. *, $p < 0.05$; **, $p < 0.01$.
(F) Blocking IFNAR1 has no effect on IFN-γ-induced activation of IRF3. RAW264.7 cells were pretreated with IgG control or IFNAR-blocking antibody (20 μg/mL) for 12 hours and then stimulated with IFN-γ (40 ng/mL) for the indicated times before immunoblot analysis of the indicated proteins.

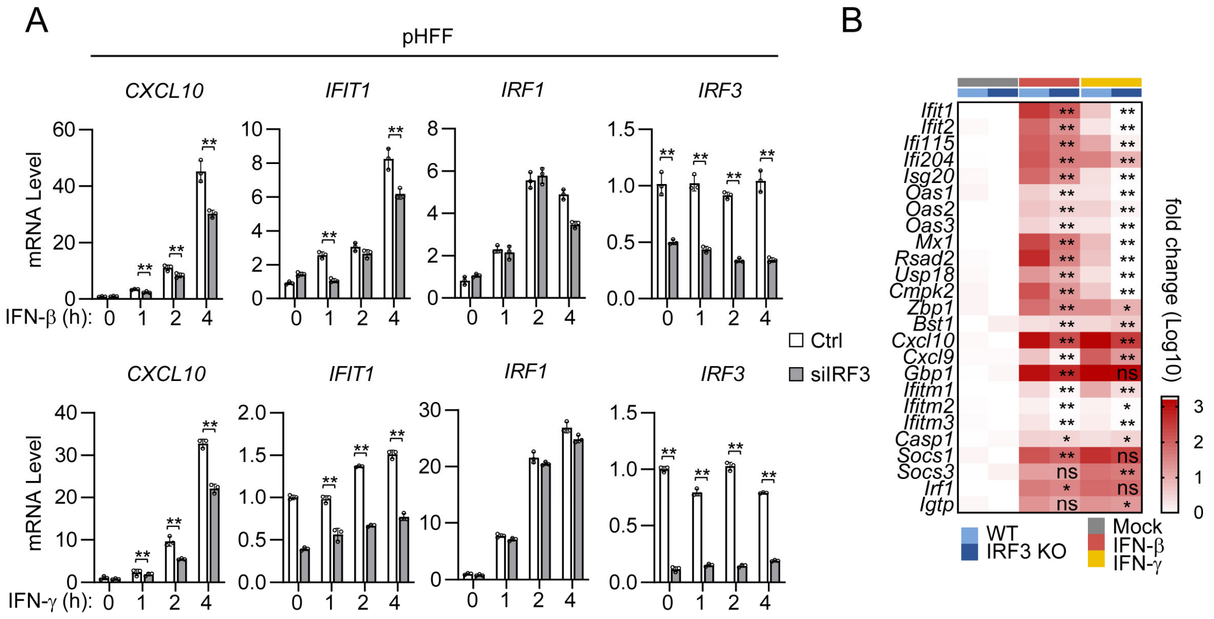

Figure S2. IFN induces IRF3-dependent transcription of downstream effector genes.
(A) Knockdown of IRF3 inhibits the IFN-β and IFN-γ-induced transcription of downstream genes. pHFF cells were transfected with control siRNA or IRF3-targeted siRNA (20 nM). 36 hours after transfection, cells were stimulated with IFN-β (20 ng/mL) or IFN-γ (20 ng/mL) for the indicated times. The mRNA abundance of the indicated genes was measured by qPCR.
(B) A heat map of qPCR of the indicated genes from WT, IRF3-deficient RAW264.7 stimulated with IFN-β (20 ng/mL) or IFN-γ (20 ng/mL) for 2 hours. The scale bar represents the fold change of mRNA abundance of each gene (log10 value).
Data are shown as mean ± SD (n=3), statistical significance was determined by unpaired two-tailed Student's *t* test. *, $p < 0.05$; **, $p < 0.01$.

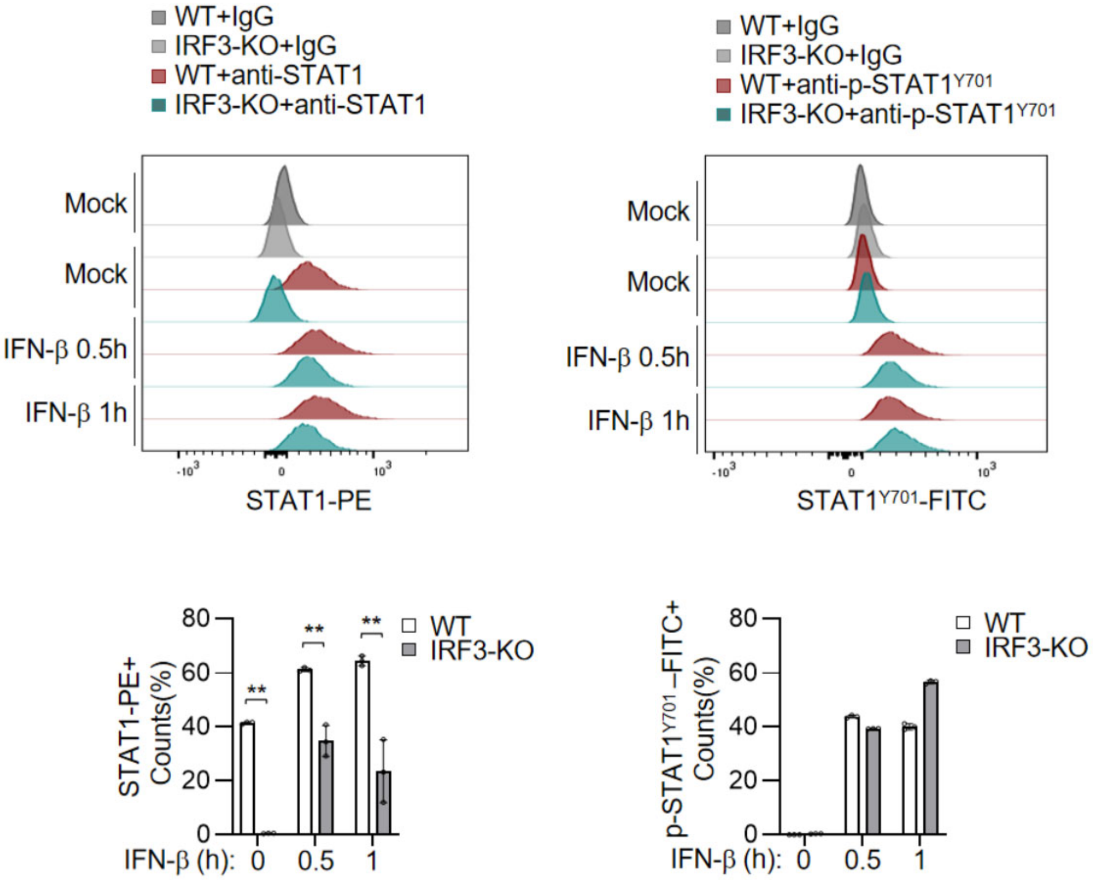

Figure S3. Knockout of IRF3 inhibited levels of STAT1 but not phosphorylated STAT1^{Y701} induced by IFN-β.

WT or IRF3-deficiency cells (1x10^7, n=3) were stimulated with IFN-β (20 ng/mL) for the indicated times. Cells were fixed with 4% formaldehyde and stained with STAT1-PE (1:200) and p-STAT1^{Y701}-FITC (1:100) antibodies before analyzed by flow cytometry. Top: Histograms representing levels of the indiacated proteins. Bottom: percentage of positive cells were calculated by STAT1+/total (left) and pSTAT1+/total (right) respectively. Data are shown as mean ± SD (n=3), statistical significance was determined by unpaired two-tailed Student's *t* test. **, $p < 0.01$.

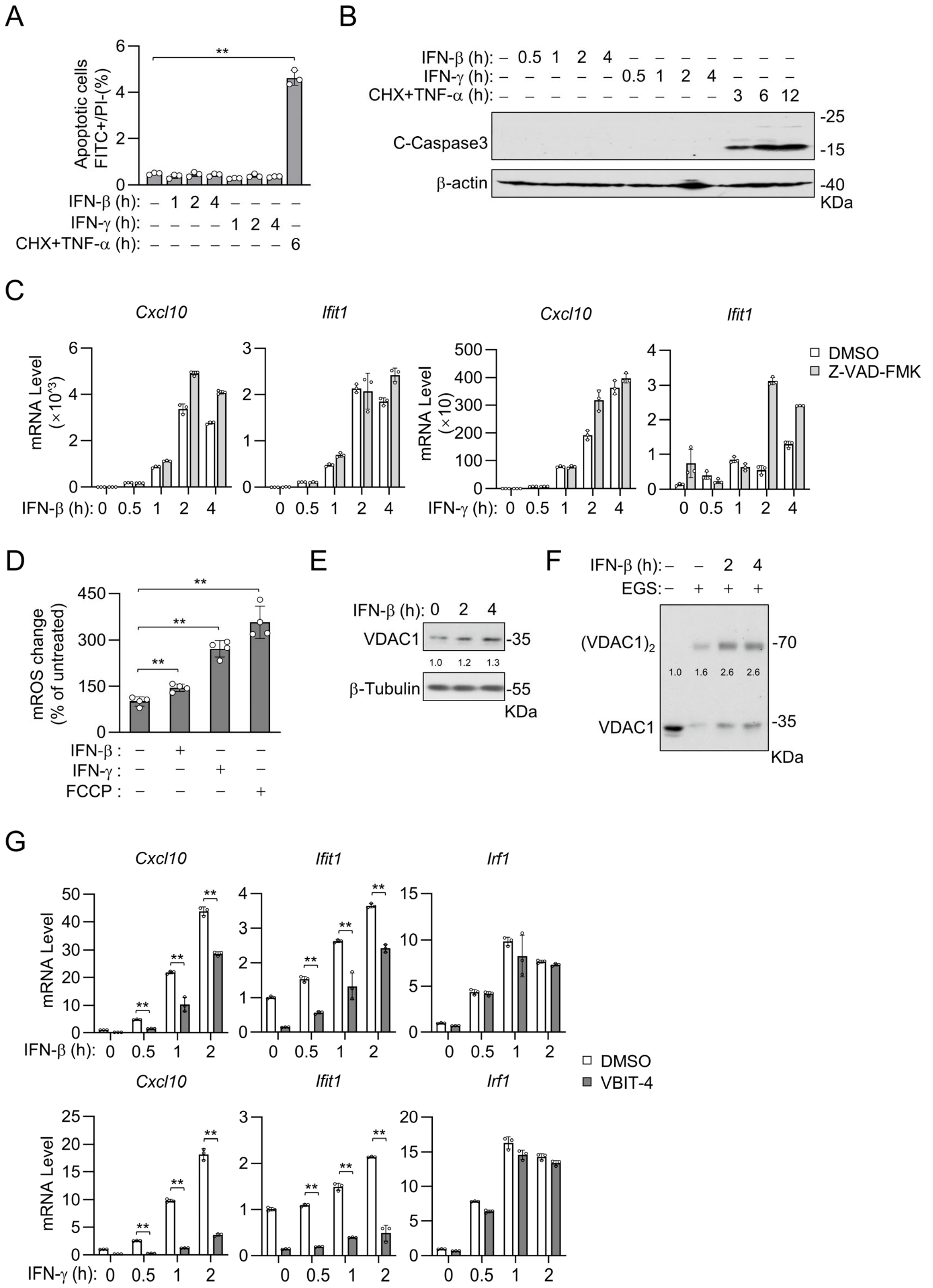

Figure S4. **IFN stimulation triggers mtDNA release by inducing mitochondrial dysfunction and formation of VDAC oligomers**

(A) Flow cytometry analysis with Annexin/PI staining was performed to evaluate the percentage of apoptotic cells in MLFs. MLFs were treated with IFN-β (40 ng/mL), IFN-γ (40 ng/mL) or CHX (50 μg/mL) and TNF-α (20 ng/mL) for the indicated times. Cells ($1x10^5$) were collected and stained with FITC-Annexin (5 μL) and PI (5 μL) for 30 min at RT before analyzed by flow cytometry. The percentage of apoptotic cells was determined by measuring the Annexin-FITC-positive and PI-negative cells.
(B) Western blot analysis of the expression of apoptosis marker cleaved-caspase3. MLFs were treated with IFN-β (40 ng/mL), IFN-γ (40 ng/mL) or CHX (50 μg/mL) and TNF-α (20 ng/mL) for the indicated times before immunoblot analysis of the indicated proteins.
(C) Inhibiting of caspase activation does not inhibit the IFN-β and IFN-γ-induced downstream genes transcription. RAW264.7 cells were unpretreated or pretreated with Z-VAD-FMK (20 μM) for 24 hours and then stimulated with IFN-β (20 ng/mL) or IFN-γ (20 ng/mL) for the indicated times. The mRNA abundance of the indicated genes was measured by qPCR.
(D) Measurement of IFN-β- or IFN-γ-induced mitoROS production. pMLFs (2×10^6) were stimulated with IFN-β (20 ng/mL) IFN-γ (20 ng/mL) or FCCP (20 μM) for the indicated times before staining with mitoSOX (5 μM) for 20 min and subsequent fluorescence detection by HCI (high content imaging) for ROS measurement.
(E) MLFs were stimulated with IFN-β (40 ng/mL) for the indicated time before analyzed by immunoblotting with the indicated antibodies. The relative quantification of the indicated proteins is shown as mean of two independent experiments.
(F) IFN-β induces the VDAC oligomerization. MLFs were stimulated with IFN-β (40 ng/mL) for the indicated times and then crosslinked with EGS (100 μM) for 30min before analyzed by immunoblotting with the indicated antibodies. The relative quantification of the indicated proteins is shown as mean of two independent experiments.
(G) MLFs were unpretreated or pretreated with VBIT-4 (10 μM) for 48 hours and then stimulated with IFN-β (20 ng/mL) or IFN-γ (20 ng/mL) for the indicated times. The mRNA abundance of the indicated genes was measured by qPCR.
Data are shown as mean ± SD (A, C, G, n=3; D, n=4), statistical significance was determined by unpaired two-tailed Student's *t* test. *, $p < 0.05$; **, $p < 0.01$.

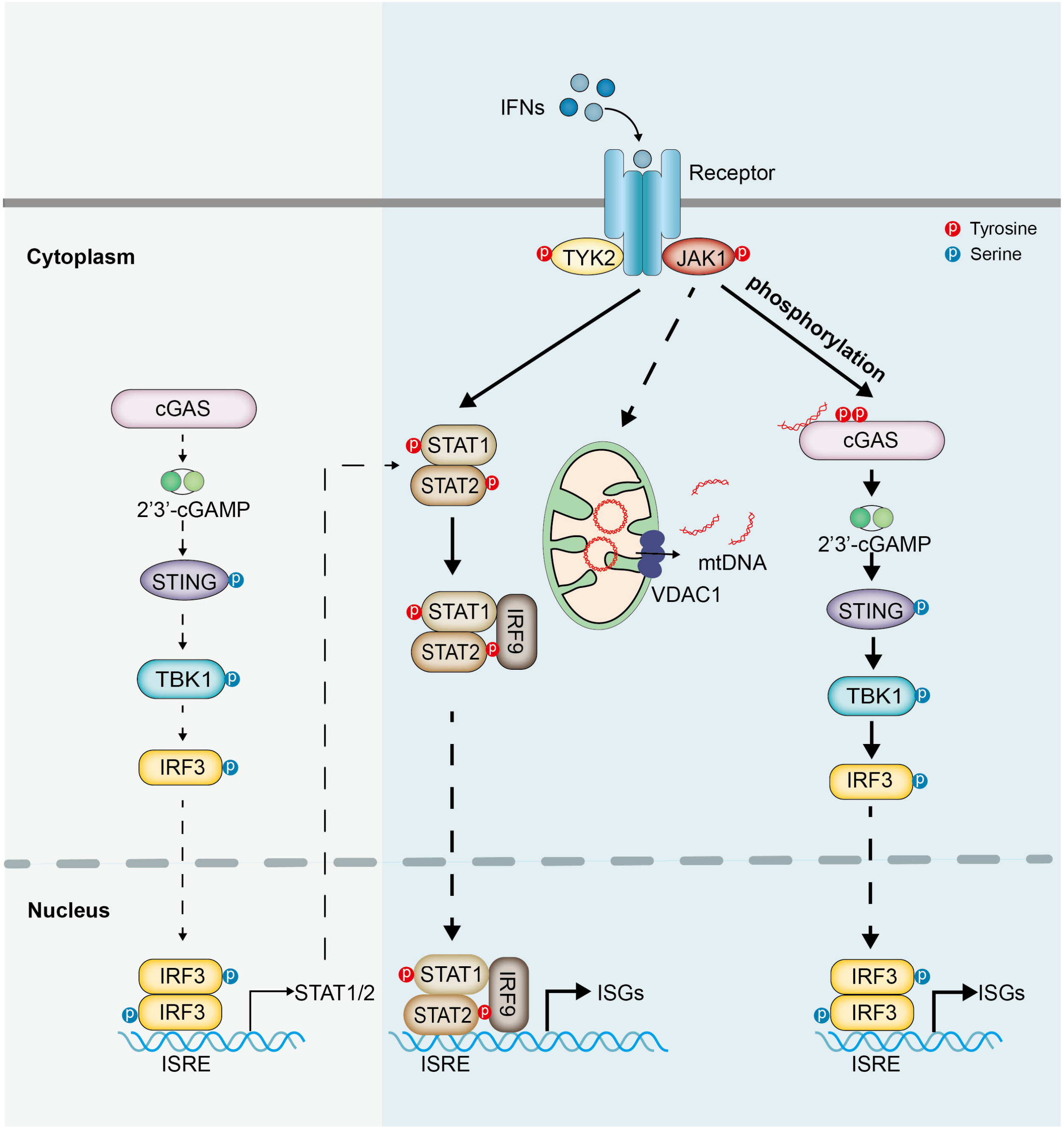

Figure S5. Illustration of the mechanism that IFN acttivates cGAS-STING-IRF3 axis.
In uninfected cells, basal expression STAT1/2 is maintained by the cGAS-STING-IRF3 axis. IFNs induce mitochondrial dysfunction and oligomerization of VDAC1, leading to release of mtDNA into the cytosol. Meanwhile, activated JAK1 phosphorylates cGAS and promotes its binding to cytosolic mtDNA, leading to activation of cGAS-STING-IRF3 pathway and induction of ISRE-containing ISGs. The JAK1-cGAS-STING-IRF3 and JAK-STAT axes coordinate to mediate the antiviral effects of interferons.

Table S1. A list of DNA oligonucleotides

HSV120	5'AGACGGTATATTTTTGCGTTATCACTGTCCCGGATTGGACACGGTCTTGT GGGATAGGCATGCCCAGAAGGCATATTGGGTTAACCCCTTTTTATTTGTG GCGGGTTTTTGGAGGACTT-3

Table S2. A list of primary antibodies used in the study.

Antibody	Supplier	Catalog No.	Appl.[a]	Usage
Mouse Flag M2 antibody clone M2	Sigma-Aldrich	F3165 Lot: SLCC4005	WB/IP	1:2000/1 μg
Mouse HA. 11 Epitope Tag antibody clone 16B12	BioLegend	901515 Lot: B29401	WB/IP	1:2000/1 μg
Mouse β-actin antibody clone ARC5115-01	ABclonal	AC026 Lot: 3503062901	WB	1:5000
Mouse β-tubulin antibody	ABclonal	AC021 Lot: 9100010002	WB	1:2000
Rabbit LMNB1 antibody	Proteintech	23498-1-AP Lot: 00080727	WB	1:1000
Rabbit VISA antibody	Santa Cruz	SC-365333 Lot: #F1419	WB	1:1000
Rabbit TBK1 antibody	Abcam	ab109735	WB	1:1000
Rabbit phosphor-TBK1 (S172) antibody	CST	5483S Lot: 13	WB	1:500
Rabbit cGAS antibody	CST	31659S Lot: 3	WB	1:1000
Rabbit STING antibody	CST	13647S Lot: 5	WB	1:1000
Rabbit phosphor-STING (S365) antibody	CST	62912S Lot: 1	WB	1:500
Rabbit IRF3 antibody	CST	4302	WB	1:1000
Rabbit phosphor-IRF3(S379) antibody	CST	79945 Lot: 1	WB	1:500
Rabbit STAT1 antibody	CST	80916	F	1:200
Rabbit STAT1 antibody	CST	9172	WB	1:1000
Rabbit phosphor-STAT1(Y701) antibody	CST	9167S Lot: 25	WB	1:1000
Rabbit phosphor-STAT1(Y701) antibody	CST	9174	F	1:200
Rabbit STAT2 antibody	CST	72604S Lot: 1	WB	1:1000
Rabbit phosphor-STAT2(Y690) antibody	CST	88410S Lot: 1	WB	1:1000
Rabbit STAT3 antibody(C-20)	Santa Cruz	sc-482	WB	1:1000
Rabbit phosphor-STAT3(Y705) antibody	CST	9145S Lot: 31	WB	1:1000
Mouse JAK1 antibody	BD transduction laboratories	610231 Lot: 7019785	WB/IP	1:1000/1μg
Rabbit phosphor-JAK1(Y1034/1035) antibody	CST	3331S Lot: 5	WB	1:500

Rabbit His-tag antibody	CST	12698S<br>Lot: 4	WB	1:1000
Mouse VDAC1 antibody	Abcam	Ab14734<br>Lot:<br>GR3401235-4	WB	1:800
Rabbit phosphotyrosine antibody, clone4G10	Millipore	05-321	WB/IP	1:1000/1μg
Rabbit Cleaved-caspase3	CST	9661	WB	1:1000
Rabbit IRF7	huabio	ET1610-89	WB	1:500
Rabbit phosphor-IRF7 (Ser437/438) antibody	CST	D6M2I	WB	1:500
Rabbit cGAS(Y215) antibody	Abclonal	AP0946	WB	1:500
Anti-mouse IFNAR1	InVivoMAb	BE0241	Blocking	1:500
Mouse-HRP	Pierce	31430	WB	1:2000
Rabbit-HRP	Pierce	31460	WB	1:2000

*CST, Cell Signaling Technology

Table S3. Primers used for site-directed mutagenesis

pMSCV-mIRF3-F-K77N/R78G-Forward	5'-GACGTGTCAACCTGG AACGGA AATTTCCGGTCAGCC-3'
pMSCV-mIRF3-F-K77N/R78G-Reverse	5'-GGCTGACCGGAAATTTCCGTTCCAGGTTGA CACGTC-3'
pMSCV-mIRF3-F-S388A/S390A- Forward	5'-GTGGACTTGCACATCGCCAACGCTCAGCCTATCTCCCTT-3'
pMSCV-mIRF3-F-S388A/S390A-Reverse	5'-AAGGGAGATAGGCTGAGCGTTGGCGATGTGCAAGTCCAC 3'
pMSCV-mIRF3-F-S378A/S379A- Forward	5'-CGGGAAGGGGGAGCCGCTGCACTGAAAACCGTG-3'
pMSCV-mIRF3-F-S378A/S379A-Reverse	5'-CACGGTTTTC AGTGCAGCGG CTCCCCCTTC CCG-3'
pLOV-cGAS-F-Δ171-174- Forward	5'-CCGGGCGGTTTTGGAGAGCCGCGATGATATCT-3'
pLOV-cGAS-F-Δ171-174-Reverse	5'-AGATATCATC GCGGCTCTCC AAAACCGCCC GG-3'
pLOV-cGAS-F-D319A- Forward	5'-GTGAAAAAATATCTGTGgctATAACCCTGGCTTTGG-3'
pLOV-cGAS-F-D319A-Reverse	5'-CCAAAGCCAGGGTTATAGCCACAGATATTTTTTCAC-3'
pLOV-cGAS-F-Y214F/Y215F- Forward	5'-CTGCTGAACACCGGGAGCTTTTTTGAGCACGTGAAGATT-3'
pLOV-cGAS-F-Y214F/Y215F-Reverse	5'-AATCTTCACGTGCTCAAAAAAGCTCCCGGTGTTCAGCAG-3'
pLOV-cGAS-F-Y214F - Forward	5'-CTGCTGAACACCGGGAGCTTTTATGAGCACGTGAAGATT-3'
pLOV-cGAS-F-Y214F - Reverse	5'-AATCTTCACGTGCTCATAAAAGCTCCCGGTGTTCAGCAG-3'
pLOV-cGAS-F-Y215F - Forward	5'-CTGCTGAACACCGGGAGCTACTTTGAGCACGTGAAGATT-3'
pLOV-cGAS-F-	5'-AATCTTCACGTGCTCAAAGTAGCTCCCGGTGTTCAGCAG-3'

Y215F - Reverse	
pLOV-cGAS-F-Y242F/Y248F/Y249F-Forward	5'-GAAGAATTTTCCAACACTCGTGCATTTTTCTTTGTGAAAT-3'
pLOV-cGAS-F-Y242F/Y248F/Y249F -Reverse	5'-ATTTCACAAAGAAAAATGCACGAGTGTTGGAAAATTCTTC-3'
pLOV-cGAS-F-Y358F- Forward	5'- GACTAAAGCCATTTTTCCTTGTACCCAAGCATGC -3'
pLOV-cGAS-F-Y358F-Reverse	5'- GCATGCTTGGGTACAAGGAAAAATGGCTTTAGTC -3'
pLOV-cGAS-F-Y415F- Forward	5'- AAACTAATGAAATTCCTTTTAGAACAGCTGA -3'
pLOV-cGAS-F-Y415F- Reverse	5'- TCAGCTGTTC TAAAAGGAAT TTCATTAGTTT -3'
pLOV-cGAS-F-Y436F- Forward	5'- GGATAAATTCTCTTCTTTTCATGTGAAAACTGCCTT -3'
pLOV-cGAS-F-Y436F-Reverse	5'-AAGGCAGTTT TCACATGAAA AGAAGAGAAT TTATCC -3'
pLOV-cGAS-F-Y470F-Forward	5'- GATAACTGCGTGACATTCTTTCTTCAGTGCCTCA-3'
pLOV-cGAS-F-Y470F-Reverse	5'-TGAGGCACTGAAGAAAGAATGTCACGCAGTTATC-3'
pLOV-cGAS-F-Y483F-Forward	5'- CAGAAAAACTTGAGAATTTTTTTATTCCTGAATTC-3'
pLOV-cGAS-F-Y483F-Reverse	5'-GAATTCAGGAATAAAAAAATTCTCAAGTTTTTCTG-3'
pLOV-cGAS-F-Y510F-Forward	5'- CAAAGCAAATTGAATTTGAAAGAAACAATGAG-3'
pLOV-cGAS-F-Y510F-Reverse	5'-CTCATTGTTTCTTTCAAATTCAATTTGCTTTG-3'

Table S4. A list of gRNA sequences.

Mouse cGAS-gRNA	5'-CGAGGCGCGGAAAGTCGTAA-3'
Mouse-STING-gRNA	5'-GCTCTTCAGCCAGACAGCAG-3'
Mouse-VISA-gRNA	5'-GTTTTGCTGTGTTGACGTTC-3'
Mouse-TBK1-gRNA	5'-CATCATGCGCGTCATAGGGG-3'
Mouse-IRF3-gRNA	5'-GAACGAGGTTCAGGATCCCG-3'
Mouse-STAT1-gRNA	5'-GGTCGCAAACGAGACATCAT-3'
Mouse-JAK1-gRNA	5'-TCCGAACCGAATCATCACTG-3'

Table S5. qPCR primers used in this study

Gbp2- Forward	5'-GGAGAGTGCTGTGCTGACTT-3'
Gbp2-Reverse	5'-GCCTCACTCTCAATTGGCCT-3'
Irf1- Forward	5'-GGGGACATTGGGATAGGCAT-3'
Irf1-Reverse	5'-GCACAAGGAATGGCCTGAAT-3'
Gapdh-Forward	5'-GAAGGGCTCATGACCACAGT-3'
Gapdh- Reverse	5'-GGATGCAGGGATGATGTTCT-3'

Ifnb1- Forward	5'-TCCGAGCAGAGATCTTCAGGAA-3'
Ifnb1-Reverse	5'-TGCAACCACCACTCATTCTGAG-3'
Ifna- Forward	5'-CCTGAGAAGAGAAGAAACACAGCC-3'
Ifna-Reverse	5'-GGCTCTCCAGACTTTCTGCTCTG-3'
Ifit1- Forward	5'-ACAGCAACCATGGGAGAGAATGCTG-3'
Ifit1-Reverse	5'-ACGTAGGCCAGGAGGTTGTGCAT-3'
Cxcl10- Forward	5'-GGTCTGAGTGGGACTCAAGG-3'
Cxcl10-Reverse	5'-GTGGCAATGATCTCAACACG-3'
Stat1- Forward	5'-GCCTCTCATTGTCACCGAAGAAC-3'
Stat1-Reverse	5'-TGGCTGACGTTGGAGATCACCA-3'
Stat2-Forward	5'-TTCTTGTTCCACCTTCGGCA-3'
Stat2-Reverse	5'-ATGCAGGGCTGGGTTTCTAC-3'
Stat3-Forward	5'-CACCTTGGATTGAGAGTCAAGAC-3'
Stat3-Reverse	5'-AGGAATCGGCTATATTGCTGGT-3'
Dloop1- Forward	5'-AATCTACCATCCTCCGTGAAACC-3'
Dloop1-Reverse	5'-TCAGTTTAGCTACCCCCAAGTTTAA-3'
Dloop2- Forward	5'-CCCTTCCCCATTTGGTCT-3'
Dloop2-Reverse	5'-TGGTTTCACGGAGGATGG-3'
Tert-Forward	5'-GCCAGCACGTTTCTCTCGTT-3'
Tert-Reverse	5'-CTAGCTCATGTGTCAAGACCCTCTT-3'
Oas1- Forward	5'-GAGGTGGAGTTTGATGTGCTGC-3'
Oas1- Reverse	5'-GTGAAGCAGGTAGAGAACTCGC-3'
Oas2- Forward	5'-CACCAAAGTCCTGAAGACCGTC-3'
Oas2- Reverse	5'-AGAGTCGTAACTCTCCAGCGAG-3'
Oas3- Forward	5'-TTCTCTGCCAGCTTCGGAAAGC-3'
Oas3- Reverse	5'-CTCTGAAGGCAGACTTGTGACC-3'
Ifitm1- Forward	5'-GCCACCACAATCAACATGCCTG-3'
Ifitm1- Reverse	5'-ACCCACCATCTTCCTGTCCCTA-3'
Ifitm2- Forward	5'-CACTCTTCTTCAACGCCTGCTG-3'
Ifitm2- Reverse	5'-GGAGCTGATATTCAGGCACTTGG-3'
Ifitm3- Forward	5'-TTCTGCTGCCTGGGCTTCATAG-3'
Ifitm3- Reverse	5'-ACCAAGGTGCTGATGTTCAGGC-3'
Rsad2- Forward	5'-GGAAGGTTTTCCAGTGCCTCCT-3'
Rsad2- Reverse	5'-ACAGGACACCTCTTTGTGACGC-3'
Zbp1- Forward	5'-GATCTACCACTCACGTCAGGAAG-3'
Zbp1- Reverse	5'-GGCAATGGAGATGTGGCTGTTG-3'
Casp1- Forward	5'-GGCACATTTCCAGGACTGACTG-3'
Casp1- Reverse	5'-GCAAGACGTGTACGAGTGGTTG-3'
Cmpk2- Forward	5'-AACTCTGCGGTGTTCCAAGACC-3'
Cmpk2- Reverse	5'-GGAACTTCCCTTTCTGGACCTC-3'

Isg20- Forward	5’-GCCATTTGGTGAAGCCAGGCTA-3’
Isg20- Reverse	5’-AGCCTGTCTGTGGACGTGTCAT-3’
Cxcl9- Forward	5’-CCTAGTGATAAGGAATGCACGATG-3’
Cxcl9- Reverse	5’-CTAGGCAGGTTTGATCTCCGTTC-3’
Mx1- Forward	5’-TGGACATTGCTACCACAGAGGC-3’
Mx1- Reverse	5’-TTGCCTTCAGCACCTCTGTCCA-3’
Bst1- Forward	5’-CGTGCTGTATGGCAAAGTTGGC-3’
Bst1- Reverse	5’-CAATAGGAGTCCACGGCGTTGT-3’
Ifi15- Forward	5’-TGGTACAGAACTGCAGCGAG-3’
Ifi15- Reverse	5’-AGCCAGAACTGGTCTTCGTG-3’
Igtp- Forward	5’-CTCATCAGCCCGTGGTCTAAA-3’
Igtp- Reverse	5’-TCACCGCCTTACCAATATCTTCA-3’
Ifi204- Forward	5’-GACAACCAAGAGCAATACACCA-3’
Ifi204- Reverse	5’-ATCAGTTTGCCCAATCCAGAAT-3’
Socs1- Forward	5’-AGTCGCCAACGGAACTGCTTCT-3’
Socs1- Reverse	5’-GTAGTGCTCCAGCAGCTCGAAA-3’
Socs3- Forward	5’-GGACCAAGAACCTACGCATCCA-3’
Socs3- Reverse	5’-CACCAGCTTGAGTACACAGTCG-3’
Ifit2- Forward	5’-CGAACTACCGTCTGGATGACTG-3
Ifit2- Reverse	5’-CTTCAACCAGCGCCATTGCTTG-3
Usp18- Forward	5’-GGAACCTGACTAAGGACCAGATC-3’
Usp18- Reverse	5’-GAGAGTGTGAGCAGTTTGCTCC-3’
ISG56- Forward	5’-TCATCAGGTCAAGGATAGTC-3
ISG56- Reverse	5’-CCACACTGTATTTGGTGTCTAGG-3
CXCL10- Forward	5’-GGTGAGAAGAGATGTCTGAATCC-3
CXCL10- Reverse	5’-GTCCATCCTTGGAAGCACTGCA-3
GAPDH- Forward	5’-GACAAGCTTCCCGTTCTCAG-3
GAPDH- Reverse	5’-GAGTCAACGGATTTGGTCGT-3
IRF3- Forward	5’-TCTGCCCTCAACCGCAAAGAAG-3
IRF3- Reverse	5’-TACTGCCTCCACCATTGGTGTC-3
IRF1- Forward	5’-GAGGAGGTGAAAGACCAGAGCA-3
IRF1- Reverse	5’-TAGCATCTCGGCTGGACTTCGA-3
VSV *L* - Forward	5’-ATTCTCTTCCGATTCCCTCG-3’
VSV *L* -Reverse	5’-CCAACTTCCCATCCATTTATGC-3’
HSV-1 *UL48*- Forward	5’-CGACCTGTTTGACTGCCTCT-3’
HSV-1 *UL48*-Reverse	5’-GACGAACATGAAGGGCTGGA-3’
SARS-CoV-2 *N* -Forward	5’- CATTGGCATGGAAGTCACAC -3’
SARS-CoV-2 *N* -Reverse	5’- TCTGCGGTAAGGCTTGAGTT -3’

Full scans of original blots for data in Figures 1-5, S1 and S4.

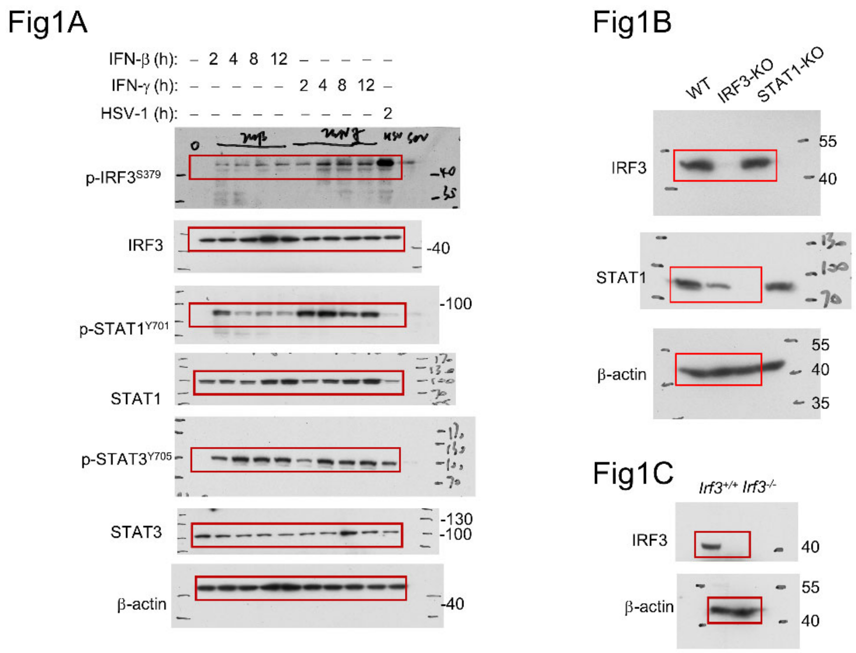

Fig2A

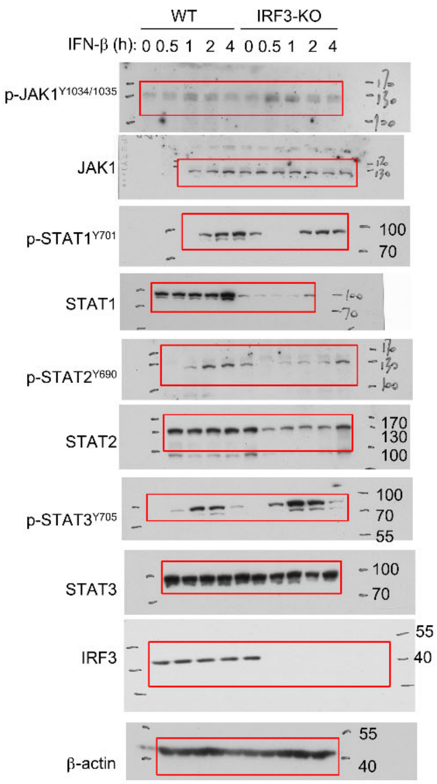

Fig2B

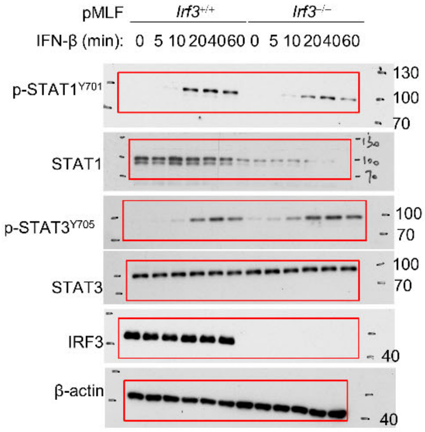

Fig2C

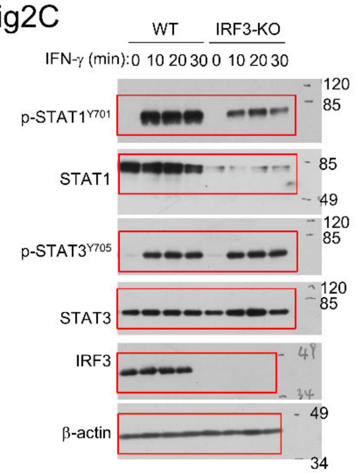

Fig2D

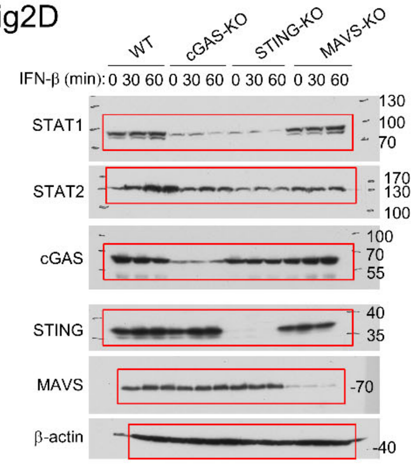

Fig2F

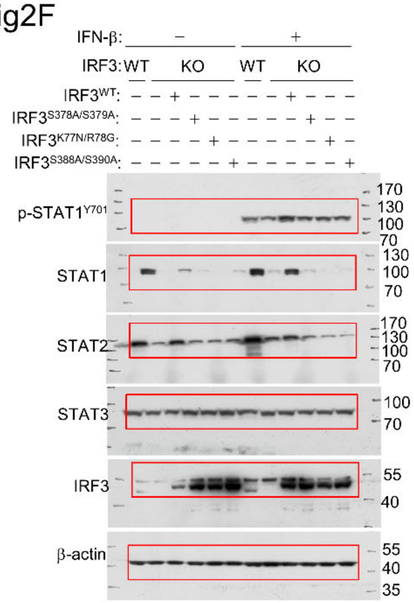

Fig3E

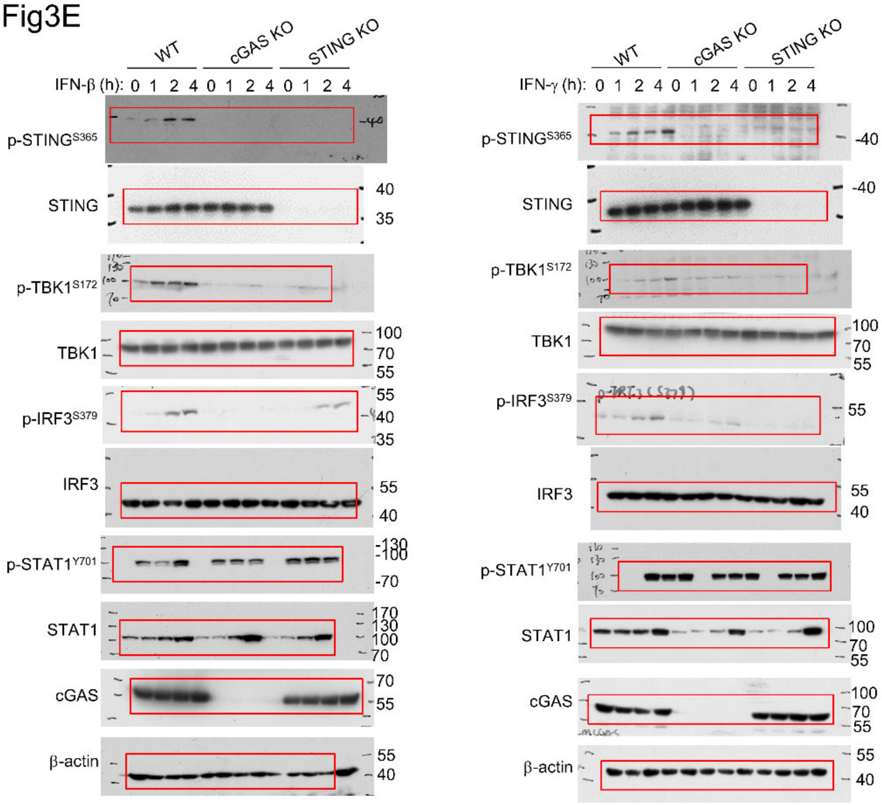

Fig4A

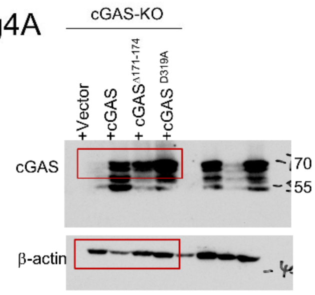

Fig4F

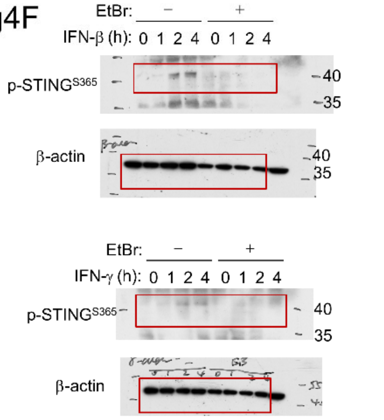

Fig5A

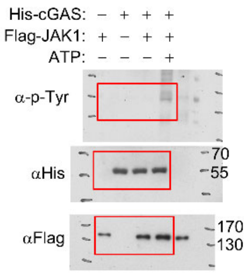

Fig5B

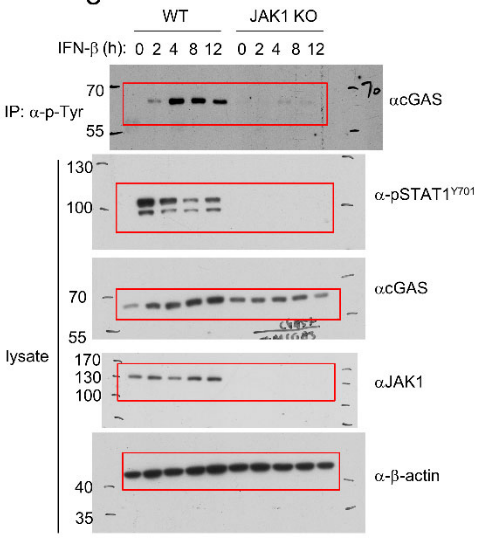

Fig5D

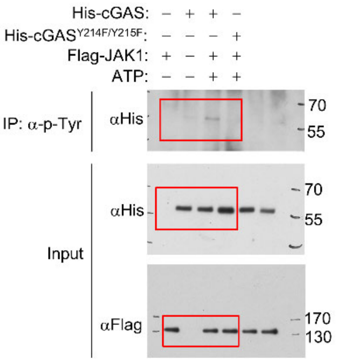

Fig5F

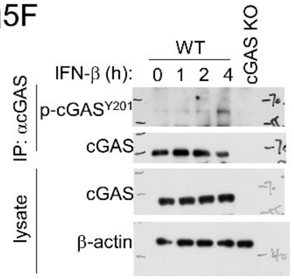

Fig5C

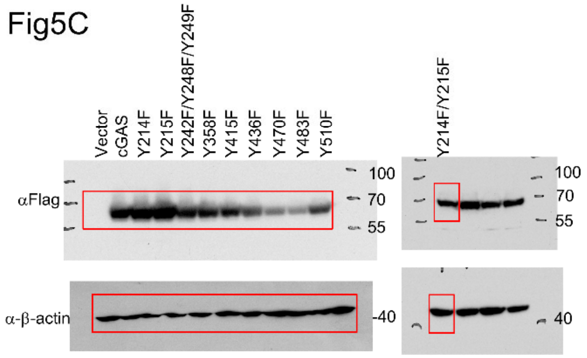

Fig5G

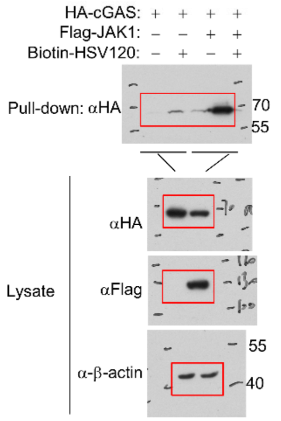

FigS1A

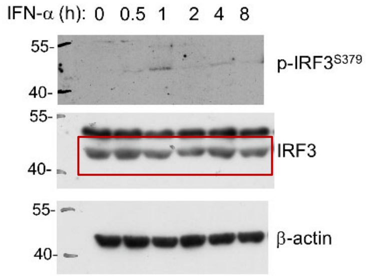

FigS1C

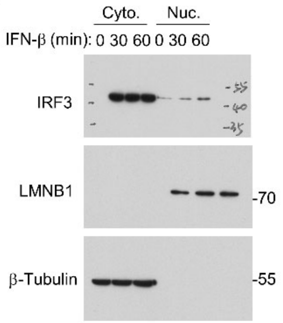

FigS1D

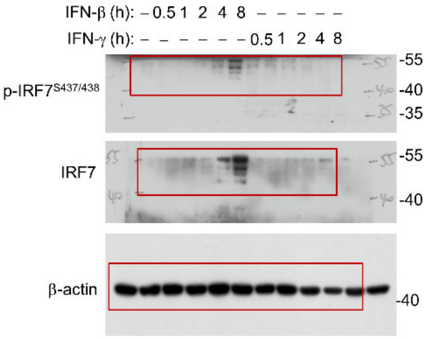

FigS1B

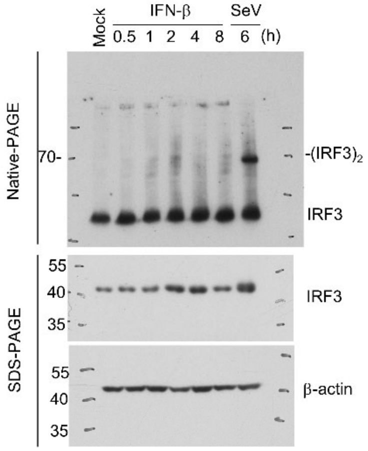

FigS1F

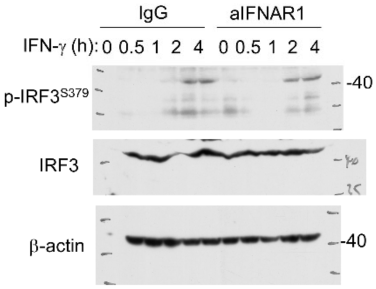

FigS4B

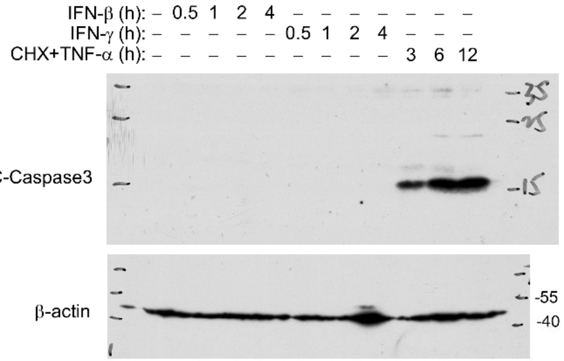

FigS4E

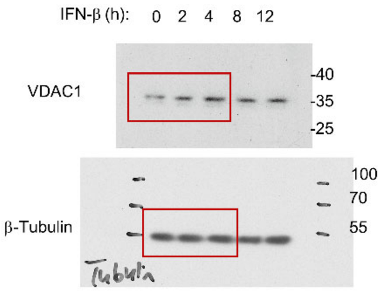

FigS4F

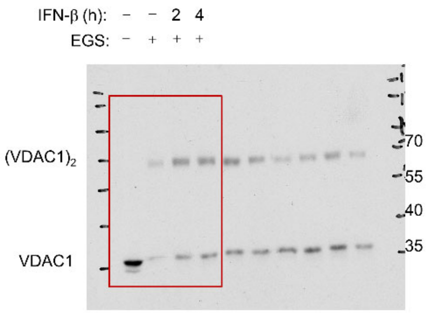

Supplement: Supplementary file 1 — Supporting Information [file ADVS-11-2308890-s001.pdf]
